# Trichothecene mycotoxin-induced ribotoxic stress activates histone gene transcription

**DOI:** 10.1007/s00204-026-04375-2

**Published:** 2026-03-31

**Authors:** Andreas F. Kolb, Vanina Popova, Linda Petrie

**Affiliations:** Nutrition, Obesity and Disease Research Theme, Rowett Institute, University of Aberdeen, Foresterhill, Aberdeen, AB25 2ZD UK

**Keywords:** Mycotoxin, Trichothecene, MAP kinase, RNA sequencing

## Abstract

Mycotoxins are secondary fungal metabolites which have toxic effects on other organisms. The trichothecenes mycotoxins deoxynivalenol (DON) and T2/HT2 are secreted by Fusarium species and are found across the globe. They are under surveillance in many jurisdictions. The main toxic effect of DON and T2/HT2 is the inhibition of protein translation by toxin binding to the peptidyl transfer centre of the large ribosomal subunit. We have characterised the effects of DON and HT2 (at IC75) on the transcriptome of the lung fibroblast cell line V79 over a time course of 15, 30, 60, 90, 120, 180 min and 24 h. The data demonstrate that the gene expression changes found at 24 h post exposure are different to those found at earlier time points. The immediate, albeit transient, effect of DON and HT2 exposure is the activation of the p38–Fos/Jun MAP kinase stress signalling pathway. This rapid response also entails a substantial activation of histone genes; a response typically associated with cell cycle entry. The activation of the p38–Fos/Jun stress pathway peaks at 90 min post exposure in response to DON and at 120 min post exposure for HT2. Gene expression changes triggered by DON exposure during the first 60 min significantly overlap with those elicited by other chemicals. In contrast, longer term exposure to DON elicits transcriptome responses which are specific to treatment with inhibitors of translation.

**Supplementary Information:**

The online version contains supplementary material available at 10.1007/s00204-026-04375-2.

## Introduction

Trichothecene mycotoxins are synthesized by fungi of the species Fusarium, Myrothecium, Stachybotrys, and Trichoderma (Gutiérrez et al. 2021). The biosynthesis process is dependent on a terpene synthase which catalyses the synthesis of the parent compound of all trichothecenes, trichodiene (Kimura et al. 1998; Nesic et al. 2014; Gutiérrez et al. 2021).

The trichothecene mycotoxins of most relevance in food and feed include deoxynivalenol (or vomitoxin), nivalenol, diacetoxyscirpenol, and T2/HT2 toxin (Yang et al. 2020; Polak-śliwińska and Paszczyk 2021; Alvito et al. 2022). Trichothecene mycotoxins mainly affect cereals, including wheat, barley, rye, oat and maize. Trichothecenes, like most mycotoxins, are chemically modified by a number of enzymes expressed in plants, bacteria and other fungi with which these organisms defend themselves against mycotoxin exposure (Lyagin and Efremenko 2019).

The trichothecene mycotoxins deoxynivalenol and T2/HT2 are under surveillance by government agencies (e.g., EFSA, FDA, FSA, State Administration of Market Regulation in China). The analysis of mycotoxins is typically carried out using immunological (e.g., lateral flow tests or ELISAs) and chemical tests (e.g., LC–MS) (Nolan et al. 2019). However, the chemical derivatives of mycotoxins are often not identified, especially by immunological tests (Gratz et al. 2013; Gratz 2017; Daud et al. 2020). These toxins are therefore often described as masked mycotoxins. While the masked mycotoxins may have reduced toxicity, e.g., due to reduced bioavailability, they may reacquire their toxicity by contact with the gut microbiome (Gratz 2017).

Exposure to trichothecene mycotoxins, like deoxynivalenol and T2/HT2, lead to reduced food intake (deoxynivalenol is known as “vomitoxin”) and impaired immune function (Pestka and Smolinski 2005; Pestka 2010; Hooft and Bureau 2021). The reduction in food intake in response to deoxynivalenol or exposure is mediated by the gut satiety peptides GLP-1, CCK and GIP (Jia et al. 2017; Yue et al. 2021). Inhibition of GLP-1 and GIP receptors by chemical antagonists attenuates the DON-induced reduction in food intake in a mouse model system (Jia et al. 2017; Yue et al. 2021).

At a molecular level the trichothecene mycotoxins deoxynivalenol and T2 lead to a reduction of protein synthesis (Bae and Pestka 2008; Pestka 2010) by binding to the peptidyl transfer centre of the large ribosomal subunit (Shifrin and Anderson 1999; Garreau De Loubresse et al. 2014; Pierron et al. 2016a). Other modes of toxicity have also been suggested, including anti-inflammatory effects (Sugiyama et al. 2016), food refusal effects (Zhou and Pestka 2015), oxidative stress (Tolosa et al. 2012; Juan-García et al. 2019), and ER stress (Shi et al. 2009). However, all or most of these effects may be interdependent and linked at a molecular level.

In order to assess the precise effects of the trichothecene mycotoxins deoxynivalenol and HT2 on the transcriptome over a time course we exposed the Chinese Hamster lung fibroblast cell line V79 to an IC75 dose of the two toxins for 15, 30, 60, 90, 120 and 180 min. We also analysed the transcriptome after an incubation of 24 h. The V79 cells were chosen as they have low levels of cytochrome P450 enzyme expression and are consequently sensitive to toxins (Doehmer 1993; Liu and Glatt 2010). They are therefore often used in toxicological research and show high sensitivity to exposure to deoxynivalenol and T2/HT2 mycotoxins (Cheli et al. 2015).

## Methods

### Cell culture

V79 Chinese hamster (*Cricetulus griseus*) lung fibroblasts (ECACC catalogue number: 86041102) and HEK293 human embryonic kidney cells (ECACC No: 85120602) were grown in DMEM (Sigma, D-6171) supplemented with 10% foetal calf serum (Sigma), 2 mM glutamine, 1mM sodium pyruvate, non essential amino acids and penicillin/streptomycin at 37 °C and 5% CO_2_ in a humidified incubator (all supplements were purchased from Sigma). To identify the IC50 doses for deoxynivalenol (DON) and HT2 mycotoxins the cells were treated with a dilution series of the toxins ranging from 2000 to 0.1 ng/ml (with 1 in 4 dilution steps). Cell viability was measured using the Cell Titre Blue reagent (Promega). Phorbol 12-myristate 13-acetate (P8139), deoxynivalenol (D0156), HT2 (T4138) and anisomycin (ANI) (A9789) were purchased from Sigma-Aldrich-Merck and dissolved in ethanol at a concentration of 2 mg/ml.

### RNA isolation

For RNA sequencing analyses V79 cells were treated with a toxin dose equivalent to the IC75 concentration values for 15 min (T15), 30 min (T30), 60 min (T60), 90 min (T90), 120 min (T120), 180 min (T180; HT2 treatment only) and 24 h (T24). The expression rates were compared to untreated cells (T0).

RNA (n = 6) was isolated using the Direct-zol RNA Mini kit (Zymo Research, R2050). RNA quality was analysed by the OD ratio 260/280 and Agilent Tapestation electrophoresis analysis. Samples with an OD 260/280 ratio above 1.8 and an RNA integrity number (RIN) above 9 were used for analysis.

### qPCR

Quantitative PCR was carried out using primer pairs (designed by Primer BLAST) and the Thermo Sybr-Green reagent. Primer sequences, amplicon sizes and annealing temperatures are shown in supplementary Table 1. Crossing points were correlated with standard curves established for each of the genes analysed. Expression of the test genes was correlated with the expression of the reference gene β-actin. Gene expression changes are presented as fold change from control values (typically vehicle treated cells). Analyses were carried out using at least 3 biological replicates with 3 technical replicates each (n ≥ 3). Toxin treatments were carried out in parallel with vehicle (ethanol) treatments.

### RNA sequencing

RNA sequencing analysis was carried out by Biomarker Technologies (BMKgene, Johann-Krane Weg 42, 48,149 Münster, Germany) for deoxynivalenol treated cells and at BGI (BGI TECH SOLUTIONS (POLAND) SP. Z O.O., Warsaw, Poland) for HT2 treated cells. 500 ng of RNA at a concentration of 50 ng/ul were supplied to BMKgene and BGI (n = 6).

FastQ files received were screened for quality using the FastQC programme. Sequences were aligned to the* Cricetulus griseus* genome (https://www.ncbi.nlm.nih.gov/datasets/genome/ GCF_000223135.1/) using Hisat2-build and samtools methods. Read counts were assessed using featureCounts.

Raw counts were correlated with total counts in the sample to derive the FPKM values (Fragments Per Kilobase of transcript per Million mapped reads). Significance values for the comparison of all counts at the different time points were analysed by a two-tailed student t-test in Excel and subsequently confirmed by one-way ANOVA in GraphPad Prism. Genes were sorted in Excel due to the significance in gene expression changes. Genes whose expression was changed with a significance value of p > 0.05 were removed from the analysis. Genes whose raw counts at treatment time-points T15 to T24 was lower than 2 were removed. Genes whose expression was undetectable at T0 (i.e., 0 raw counts) were also removed.

### Western blot analysis

Western blot analysis was carried out as described (Szymanowska et al. 2009). Antibodies directed against total p38 (Cell Signalling Technology catalogue number #9212), phospho-p38 (#9211), Jun (#9165) and phospho-Jun (#3270) and the horse-radish peroxidase linked goat-anti-rabbit IgG secondary antibody (#7074) were all obtained from Cell Signalling Technology. Western blot signals were quantified using the ImageJ plot profile and wand tracing tool to determine the area under the curve.

### Pathway analysis

Pathways analysis of transcriptome datasets was carried out using the G-Profiler software (Reimand et al. 2019) available at: https://biit.cs.ut.ee/gprofiler/gost and the EnrichR platform (Kuleshov et al. 2016) available at: https://maayanlab.cloud/Enrichr/. Venn diagram analysis was carried out with an online tool available at: https://bioinformatics.psb.ugent.be/webtools/Venn/.

## Results

Trichothecene mycotoxins are produced by fungi of the species Fusarium, Myrothecium, Stachybotrys, and Trichoderma (Gutiérrez et al. 2021). They typically bind to the peptidyl transfer centre of the large ribosomal subunit (Garreau De Loubresse et al. 2014). The interference with the extension of nascent protein chains activates a ribotoxic stress response (Bae and Pestka 2008; He et al. 2012), mediated by the stress MAP kinase ZAK alpha (Moon and Pestka 2003; Vind et al. 2020b, a). The outcomes of this stress response on the wider physiology of the mammalian cells are not entirely clear.

### Sensitivity of V79 cells to trichothecene mycotoxins

**Fig. 1:**
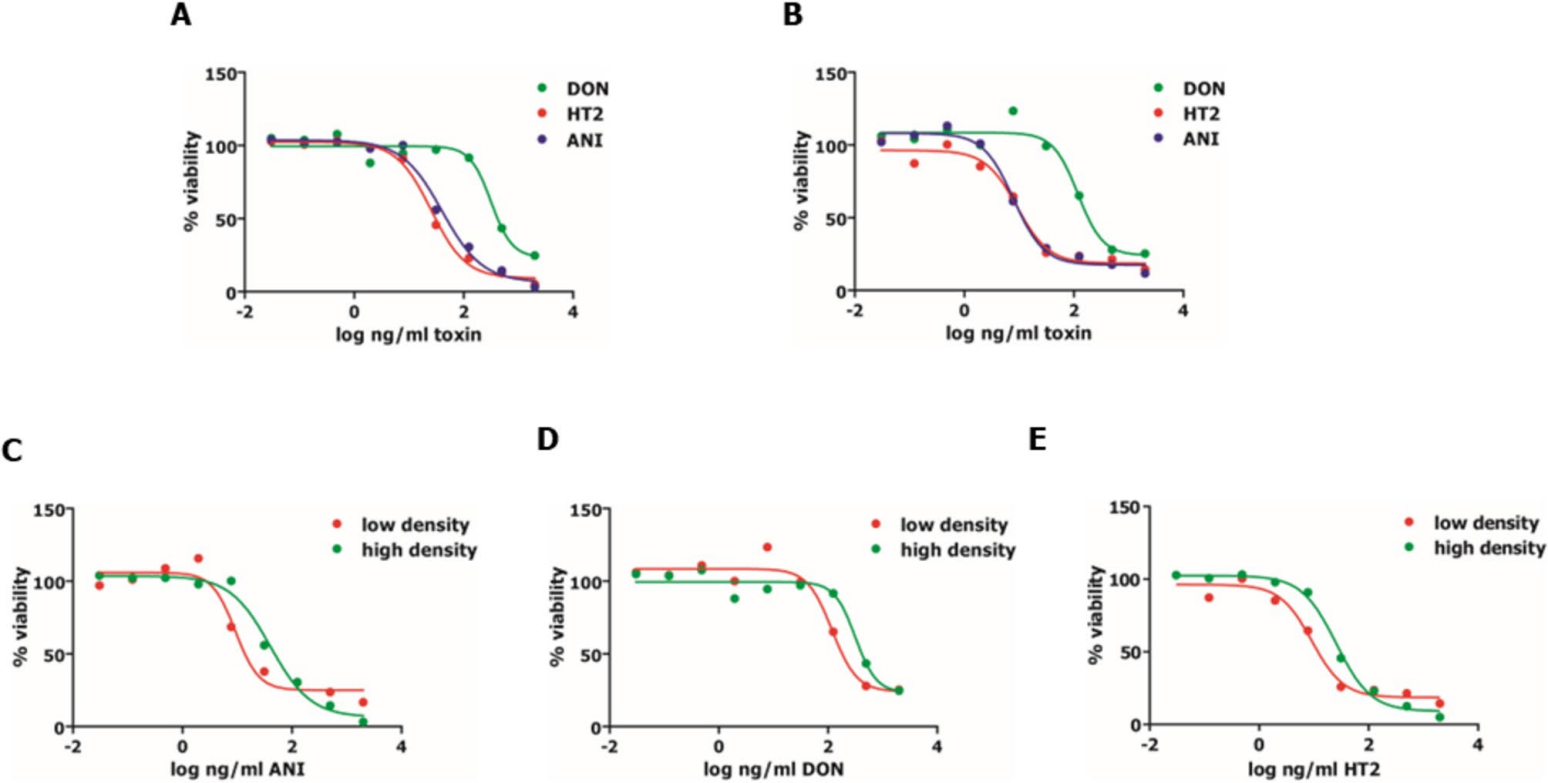
Dose response curves of cell viability in response to mycotoxin treatment in V79 Chinese Hamster lung fibroblasts. **Panel A** Dose response of V79 cells at 70% confluence to a 24 h treatment with deoxynivalenol (DON), HT2, or anisomycin (ANI). **Panel B** Dose response of V79 cells at 30% confluence to a 24 h treatment with deoxynivalenol (DON), HT2, or anisomycin (ANI). **Panel C** Comparison of deoxynivalenol treatment on the viability of V79 cells at 30% confluence (low density) and 70% confluence (high density). **Panel D** Comparison of HT2 treatment on the viability of V79 cells at 30% confluence (low density) and 70% confluence (high density). **Panel E** Comparison of anisomycin treatment on the viability of V79 cells at 30% confluence (low density) and 70% confluence (high density)

Several transcriptome analyses were carried out in the past, but typically these assessed the transcriptome changes after longer exposure (4–24 h) of cells to trichothecene mycotoxins (Hochstenbach et al. 2010; Pierron et al. 2016b; Alassane-Kpembi et al. 2017; Kalt et al. 2019; He et al. 2021; Tremblay-Franco et al. 2021). We therefore set out to characterise the transcriptome response of V79 Chinese Hamster lung fibroblasts to the trichothecene mycotoxins deoxynivalenol (DON) and T2/HT2 (supplementary Fig. 1). In all experiments the more stable T2 metabolite HT2 was used. As a first step the impact of DON and T2/HT2 (alongside the cancer drug anisomycin which shares a common biochemical target with DON and HT2) on the viability ofV79 cells were tested over a period of 24 h (Fig. 1). The values for the IC25, IC50 and IC75 concentrations were established (supplementary Table 2) after exposure of V79 cells to the 3 toxins at concentrations ranging from 2000 to 0.012 ng/ml (n = 8). The data demonstrate that DON is less toxic than both, HT2 and anisomycin. HT2 and anisomycin show almost identical IC25, IC50 and IC75 concentrations. Cell confluence impacts the effect of DON, HT2 and anisomycin on cell viability with less confluent cells showing a higher sensitivity of mycotoxin exposure (Fig. 1 and supplementary Table 2).

In order to assess how quickly V79 cells respond to treatment with DON, viability was tested using the Cell Titre Blue assay after exposure of V79 cells to DON for 2 h, 4 h, 6 h, 12 h and 24 h (n = 4). Cells were exposed to DON concentrations ranging from 2000 to 0.012 ng/ml. After 2 h no loss of viability is detectable at any DON concentration (supplementary Fig. 2a). A significant impairment of cell viability is detectable after a 4 h exposure of V79 cells to 2000 ng/ml or 500 ng/ml. A DON concentration of 125 ng/ml elicits detectable changes in cell viability after 12 h of treatment (supplementary Fig. 2b).

**Fig. 2:**
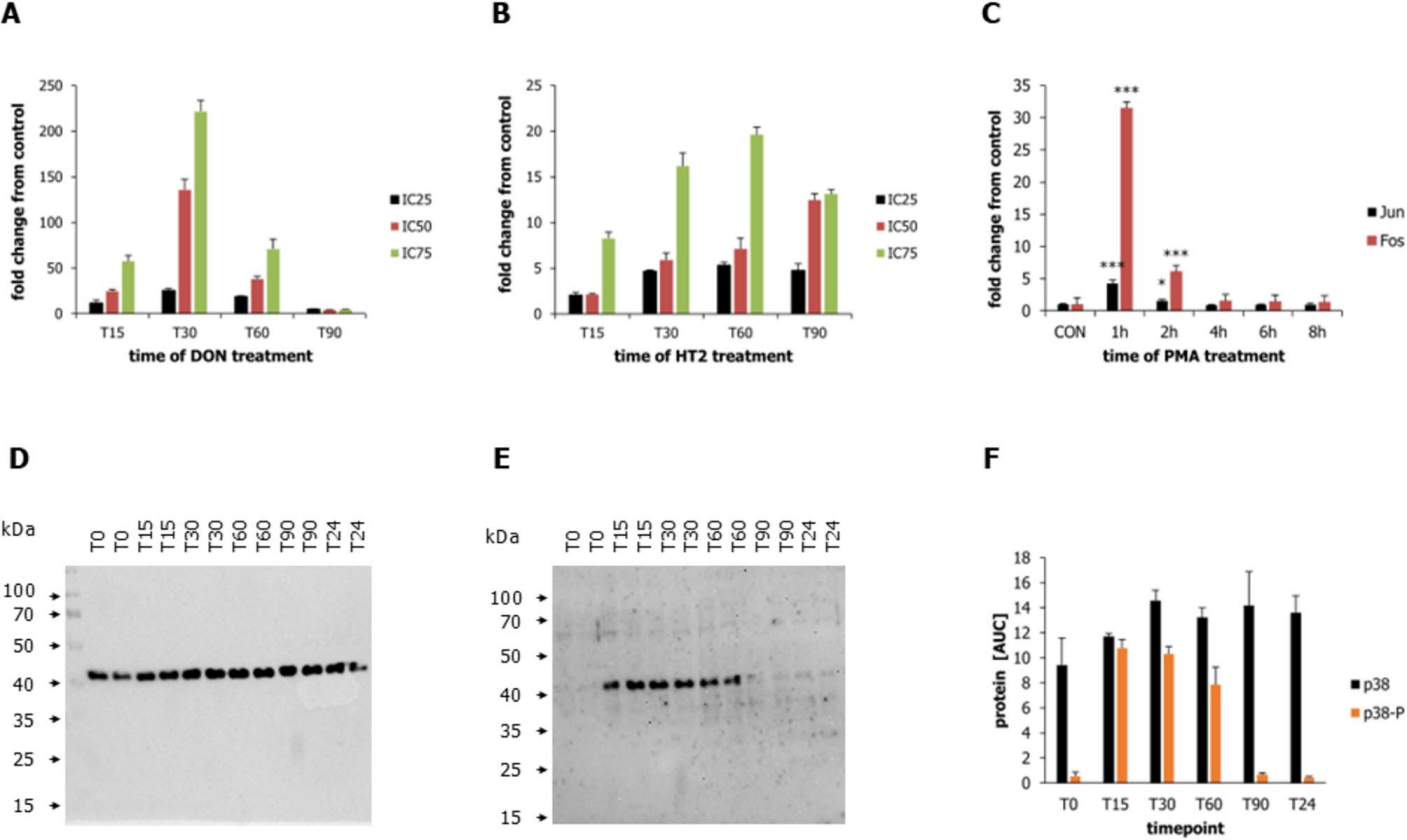
Treatment of V79 cells with IC25, IC50 and IC75 doses of the trichothecene mycotoxins deoxynivalenol and HT2, and growth promoter phorbol 12-myristate 13-acetate (PMA). Expression of the stress response marker genes c-Fos, c-Jun and the reference gene β-actin were measured by qPCR. Expression of c-Fos and c-Jun was correlated with β-actin. The fold change of c-Fos expression relative to untreated cells is shown. **Panel A** Fold change of c-Fos expression in response to deoxynivalenol (DON) treatment for 15 min. (T15), 30 min. (T30), 60 min. (T60), and 90 min. (T90) (n = 1). **Panel B** Fold change of c-Fos expression in response to HT2 treatment for 15 min. (T15), 30 min. (T30), 60 min. (T60), and 90 min. (T90) (n = 1). **Panel C** Fold change of c-Fos and c-Jun expression in response to PMA treatment for 1 h, 2 h, 4 h, 6 h and 8 h (n = 2). **Panels D** and **E** Western blot analysis of total cell extracts derived from untreated V79 cells (T0) or V79 cells treated with deoxynivalenol at the IC50 concentration for 15 (T15), 30 (T30), 60 (T60) and 90 (T90) minutes and 24 h (T24). The signal for total p38 protein (**Panel D**) and phosphorylated p38 protein (**Panel E**) are shown (n = 2). **Panel F:** Western blot signals quantified from TIF files using ImageJ shown as area under the curve [AUC]

The expression of the c-Fos gene, a major component of the ribotoxic stress response was analysed in V79 cells exposed to IC25, IC50 and IC75 concentrations of DON and HT2 (Fig. 2). Unsurprisingly, Fos expression is activated more substantially by higher toxin concentrations (e.g., IC75). In V79 cells a peak response of Fos expression is reached after 30 min of DON exposure, whereas this peak is only reached after 60 min in response to HT2 exposure (Fig. 2a, b). The expression response of Fos and Jun to DON and HT2 treatment mirrors the response observed after treatment of V79 cells with the growth promoter phorbol 12-myristate 13-acetate (PMA) (Fig. 2c). The expression of the immediate early response genes c-Jun and c-Fos are strongly increased after 1 h of treatment, but expression falls back to pre-treatment levels after 4 h (Fig. 2c). The expression of c-Fos in response to DON exposure mirrors the phosphorylation of the MAP kinase p38 as detected by Western blot analysis (Fig. 2d, e). The data shown in Fig. 2 demonstrate that V79 cells follow the same response pattern of immediate early gene expression seen in other cell types after treatment with DON (Bae and Pestka 2008) or anisomycin (Sung et al. 2023).

### RNAseq analysis of V79 cells exposed to trichothecene mycotoxins

RNA was isolated from DON and HT2 treated V79 cells after exposure to the mycotoxins at a concentration of IC75 (50 ng/ml for HT2 and 500 ng/ml for DON) for 15, 30, 60, 90, 120, 180 min and 24 h (timepoints: T15, T30, T60, T90, T120, T180, and T24, respectively). RNA was analysed by RNA sequencing. Counts were determined for each transcript. Genes in which expression of the gene (as measured by counts) in one of the timepoints was below 2 across the 6 replicates were eliminated from the analysis.

The top 50 genes expressed in V79 cells (after DON treatment) by relative RNA sequencing counts (FPKM; Fragments Per Kilobase Million) are shown in supplementary Table 3. The majority of the highly expressed genes encode ribosomal proteins, mitochondrial proteins and key structural proteins (like β-actin) and enzymes (like GAPDH). The expression of most of these genes is not changed significantly (p < 0.05) and substantially (i.e., more than twofold) in response to DON exposure.

**Fig. 3:**
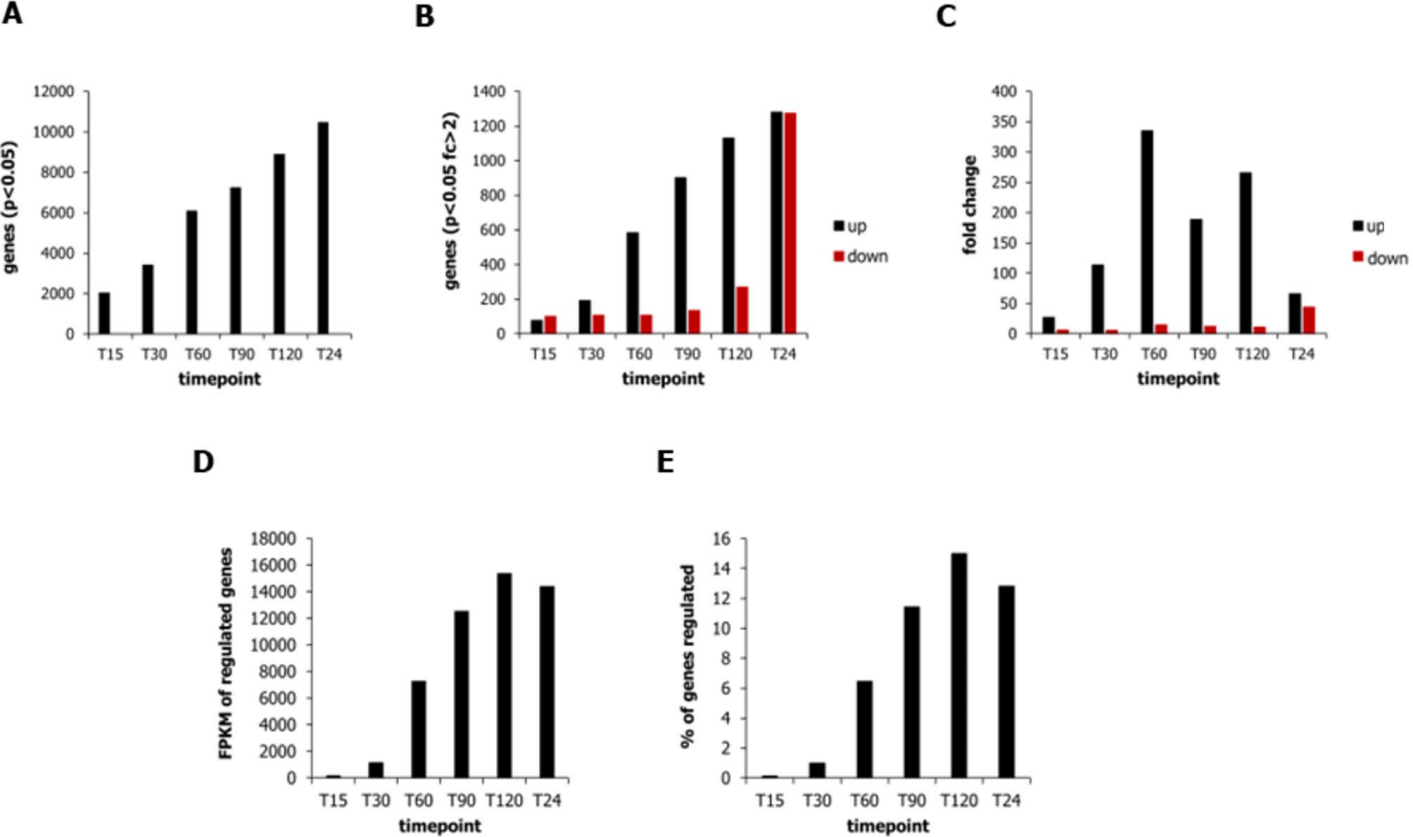
Time course of gene expression changes in V79 cells in response to deoxynivalenol (DON) exposure. **Panel A** Number of gene expression changes relative to control cells with a p-value of < 0.05 (student t-test) at timepoints T15 (15 min.), T30 (30 min.), T60 (60 min.), T90 (90 min.), T120 (120 min.) and T24 (24 h). **Panel B** Number of gene expression changed with a p value of < 0.05 and a fold change of > 2 at the different timepoints. Genes up-regulated (up) and down-regulated (down) in response to DON exposure are shown separately. **Panel C** Maximum fold change of gene expression for up-regulated (up) and down-regulated (down) genes at the different timepoints. **Panel D** Maximum expression levels (in FPKM) of the genes changed > 2, p < 0.05 at the different time points. **Panel E** FPKM of genes regulated significantly (p < 0.05) and substantively (> twofold change of expression) relative to FPKM of the 50 most highly expressed genes, shown as percentage for the different experimental time points

Statistically significant gene expression changes (p < 0.05, as analysed by a two tailed t-test) in response to DON exposure were analysed for each time point relative to timepoint T0, i.e., vehicle treated cells. The number of genes significantly changed between untreated and treated cells increased throughout the incubation phase (Fig. 3a). Most genes whose expression was changed significantly (p < 0.05) were only changed marginally (fold change < 2). Around 10% of the changed genes showed expression changes higher than twofold (Fig. 3b). During the first 2 h of mycotoxin exposure most gene expression changes consisted of activated rather than reduced gene transcription (Fig. 3b). The highest fold changes of gene expression were found during the early phase of mycotoxin exposure (between the time points T60 and T90) in which some genes were activated by up to 300-fold (Fig. 3c).

The top 50 genes regulated by DON exposure were analysed for their expression rate (in FPKM). The expression rate of the mycotoxin responsive genes which were changed significantly (p < 0.05) and substantially (i.e., more than twofold) increased over the incubation period (Fig. 3d). When the total counts/FPKM of the top 50 regulated genes is compared to the FPKM of the 50 most expressed genes in the RNAseq experiment, a steady increase in the expression of DON responsive genes is observed (Fig. 3e). This means that, e.g., at timepoint T120 the top 50 DON responsive genes show a combined FPKM count of 15% of the FPKM count of the top 50 expressed genes. This percentage remains high at time point T24 (24 h). This demonstrates that the transcriptional response to DON exposure is rapidly ramped up and then remains high even after 24 h of exposure. However, the genes regulated at T24 are different to those regulated at earlier time points of DON exposure.

The genes which show the highest fold change increase of gene expression are shown in supplementary Table 4. The genes are shown for all 6 comparisons of DON treatment of V79 cells (T15 vs T0, T30 vs T0, T60 vs T0, T90 vs T0, T120 vs T0, and T24 vs T0). The genes encoding cell stress response transcription factors (including Fos, Jun, Egr1 and Egr2) are marked in green. Genes encoding histone genes are marked in blue. The table demonstrates that histone genes are highly responsive to exposure of V79 cells to DON. The columns showing the fold change in gene expression were coloured using the conditional formatting tool in Excel. This demonstrates that the highest increases in gene expression are observed at timepoints T60, T90 and T120 (see also Fig. 3c). The genes which show the highest fold change decrease of gene expression are shown in supplementary Table 5.

The genes significantly and substantially increased by DON exposure were also sorted by FPKM counts and are shown in supplementary Table 6. The most highly expressed genes (of those regulated by more than twofold and with a significance of *p* < 0.05) appear at the top of the table. The fold change of expression relative to timepoint T0 is also shown. A comparison of supplementary Table 6 and supplementary Table 4 demonstrates that stress response transcription factors Fos, Jun and Egr1 (marked in green) are among the top 50 genes in both tables. The same is true for several histone genes (marked in blue). This indicates that these genes are highly responsive to DON exposure and expressed (at the RNA level) at a substantive rate. The expression data obtained by RNA sequencing (n = 6) were confirmed by qPCR analysis of the expression of Fos, Jun, ATF3, Egr1 and H1.2 (n = 4) (supplementary Fig. 3). The qPCR data confirm the data obtained by RNA sequencing.

**Fig. 4:**
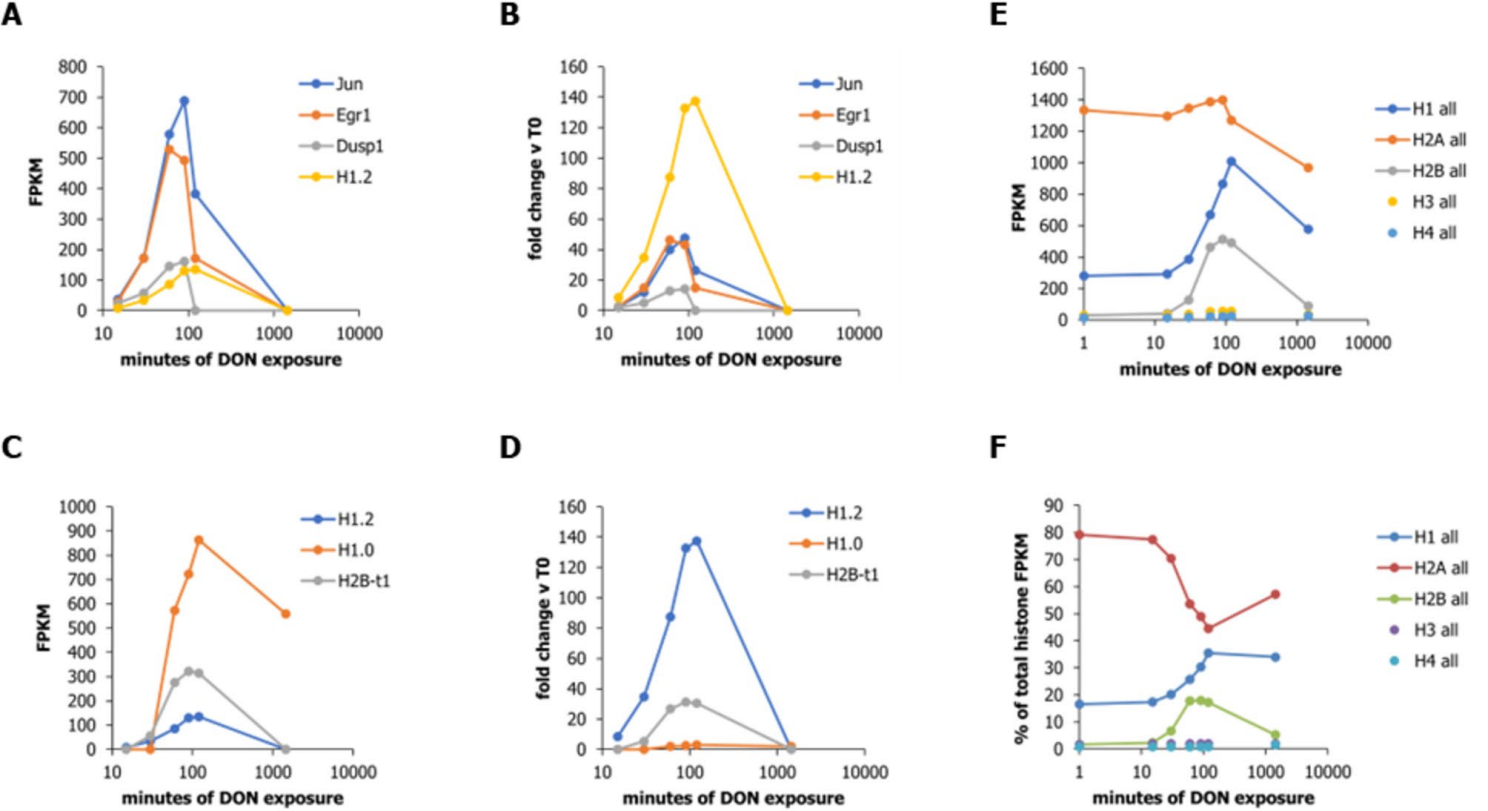
Expression of key regulated genes over time (n = 6). **Panel A** Expression of key regulated genes (transcription factors Jun, Egr1 and Dusp1, and histone H1.2) over the incubation period shown as FPKM (as a readout of expression). Note that a maximum of expression is reached at timepoint T90 (90 min of DON exposure). **Panel B** Fold change of gene expression of key regulated genes (transcription factors Jun, Egr1 and Dusp1, and histone H1.2) over the incubation period. **Panel C** Expression of key regulated histone genes (H1.2, H1.0 and H2B-t1) over the incubation period shown as FPKM. **Panel D** Fold change of gene expression of key regulated histone genes (H1.2, H1.0, H2B-t1) over the incubation period. **Panel E** Total counts (FPKM) of histone 1 (H1 all), histone H2A (H2A all), histone H2B (H2B all), histone 3 (H3 all) and histone 4 (H4 all) genes. Note that histone H2A and histone 1 are the predominantly expressed histone genes. Histone H2A gene expression reduced in response to DON exposure at T24, while histone 1 gene expression increases in response to short term DON exposure. The biggest increase in response to DON exposure is seen for histone H2B genes. **Panel F** Expression of histone 1 (H1 all), histone H2A (H2A all), histone H2B (H2B all), histone 3 (H3 all) and histone 4 (H4 all) genes over time shown as percentage of total histone gene expression

The expression of the several representative genes which are highly activated in response to DON treatment of V79 cells is shown in graphical form Fig. 4a. The expression of Jun, Egr1 and Dusp1 peaks at time point T120, for both FPKM values (Fig. 4a) and fold change of gene expression relative to control values (expression timepoint T0) (Fig. 4b). The same is true for the histones H1.2, H1.0 and H2B-t1 (Fig. 4c, d) which reflect the expression changes of numerous histone genes (supplementary Table 4). Genes which were downregulated by exposure of V79 cells to DON are shown in supplementary Tables 5 and 7. Supplementary Table 7 demonstrates that the expression rates of the genes downregulated in response to DON treatment are typically low (FPKM values below 3 for timepoints T15, T30 and T60). In contrast expression rates of the genes upregulated by DON exposure are much higher at these time points (up to FPKM above 550 for timepoint T60) (supplementary Table 6).

Expression changes observed in V79 cells exposed to the IC75 concentration of HT2 (50 ng/ml) largely mimic the changes seen in response to treatment with the IC75 concentration of DON (500 ng/ml). However, the response of cells to HT2 treatment is delayed by around 30 min (supplementary Fig. 4a and b). The peak of stress transcription factor gene expression (Fos, Jun, Egr1) occurs at around 120 min after addition of HT2, whereas it occurs 90 min after addition of DON. This may indicate that the uptake of HT2 into cells is slower than that of DON.

### Changes in histone gene transcription

Histones represent the group of genes that is most responsive to DON exposure. Of the 50 most upregulated genes at time point T15 (15 min of DON exposure) 11 are histone genes (supplementary Table 4). At time points T30, T60, T90 and T120 most upregulated genes are histones (35, 31, 27 and 27 of the 50 most upregulated genes, respectively; supplementary Table 4). Even after 24 h of DON exposure 8 of the 50 most upregulated genes are histone genes. Different classes of histone genes show distinct responses to DON treatment. Histone 1 gene expression increases steadily during the acute response to DON treatment at timepoints T30 to T120 (Fig. 4e). Overall histone 1 gene expression is increased fivefold over control levels at timepoint T120. The main H1 histone genes are H1.2 and H1.0 (supplementary Fig. 5a). Expression of the H2B genes increases around 20-fold from untreated cells to timepoint T90 (Fig. 4e, supplementary Fig. 5d). The expression of H2A histone genes overall remains largely unchanged during DON exposure of V79 cells (Fig. 4e). Nevertheless, the composition of H2A genes which contribute to the overall H2A expression levels does change (supplementary Fig. 5c). Histones H3 and H4 genes are expressed at a low level compared to histone H1, H2A and H2B genes (Fig. 4e) and increase by around twofold overall in response to DON treatment. However, individual histone 3 and histone 4 genes are strongly activated as part of the overall stress response induced by DON (Fig. 4f, supplementary Fig. 5b and e).

The predominant histone genes expressed in control cells are H2A histones. During the exposure to DON the relative expression of H2A histone is progressively reduced (supplementary Fig. 6a–g). Concomitantly the expression of H1 histones increases in response to DON exposure. The changes in H2A and H1 gene expression persist even after 24 h of DON exposure. In contrast expression of H2B histones is temporarily increased during the immediate early response gene activation. However, H2A expression returns to pre-exposure levels after 24 h of DON treatment (supplementary Fig. 6).

### Pathway analysis of transcriptional responses to trichothecene mycotoxins

The significantly (p < 0.05) and substantially upregulated genes (fold change > 2) were analysed using the pathway analysis software G-Profiler (Reimand et al. 2019). Supplementary Table 8 shows the main pathways detected as significantly changed at all timepoints of the analysis are DNA binding (GO:0003677) and binding (GO:0005488) (both marked in blue). MAP kinase activity (GO:0017017) is a significantly upregulated pathway at timepoints T60, T90 and T120. No significantly changed pathways were detected at timepoint T15. A graphical representation of these pathways is shown in supplementary Fig. 7. The number of pathways significantly changed in response to DON treatment reaches a peak at timepoints T90 and T120 (supplementary Fig. 7a). The significance of the pathway changes, however, reaches a maximum at time point T24 (see -log10 of p-value in supplementary Fig. 7b). Significantly and substantially downregulated genes (supplementary Tables 5 and 7) do not show any significantly changed pathways for most of the timepoints, with the exception of T120 (9 pathways) and T24 (1 pathway significantly changed) (data not shown).

Pathway analysis via the EnrichR software suite (https://maayanlab.cloud/Enrichr/) enabled a comparison of the transcriptome changes observed in response to DON treatment with related transcriptome changes induced by gene inactivation (via CRISPR-Cas9) and chemical treatment in the LINCS 1000 database (Subramanian et al. 2017; Evangelista et al. 2023).

These comparisons indicate significant overlaps between transcriptional programs induced by DON treatment and specific gene knockouts (supplementary Table 9; supplementary Fig. 8a). These affect the MAP kinase signalling pathway (MAP3K8, MAP4K1) and regulators of cell cycle progression and apoptosis (CHEK1, PDCD10). Most of these significant transcriptome similarities are found for genes upregulated by DON exposure (supplementary Fig. 8a, b). However, after 24 h of DON exposure the most significant similarities are found for genes downregulated by DON exposure (supplementary Fig. 8a, c). These similarities mainly affect proteins associated with ribosome function (supplementary Table 9).

Significant overlaps of transcriptome alterations were also found between DON exposure and several drugs and toxins (for which transcriptome data are available in the LINCS 1000 database). Unsurprisingly these similarities include several known translation inhibitors, most prominently, anisomycin which binds to the same site of the ribosomal peptidyl transfer centre as DON (Garreau De Loubresse et al. 2014) (supplementary Table 10). However, during the early stages after DON exposure the induced transcriptome changes also show similarity to other drugs activating the immediate early gene stress response (e.g., okadaic acid, a phosphatase inhibitor, or Cercosporin, an activator of oxidative stress). However, after 24 h of DON exposure the transcriptome changes show similarities mostly to those induced by other inhibitors of translation (7 out of the 10) (supplementary Table 10, supplementary Fig. 8d–f).

Overall, these pathway analyses demonstrate that the short-term transcription changes (within two hours after toxin exposure) induced by DON exposure affect general stress response pathways, while longer term effects (24 h after toxin exposure) affect pathways related to ribosome function.

### Protein changes induced by DON exposure

**Fig. 5:**
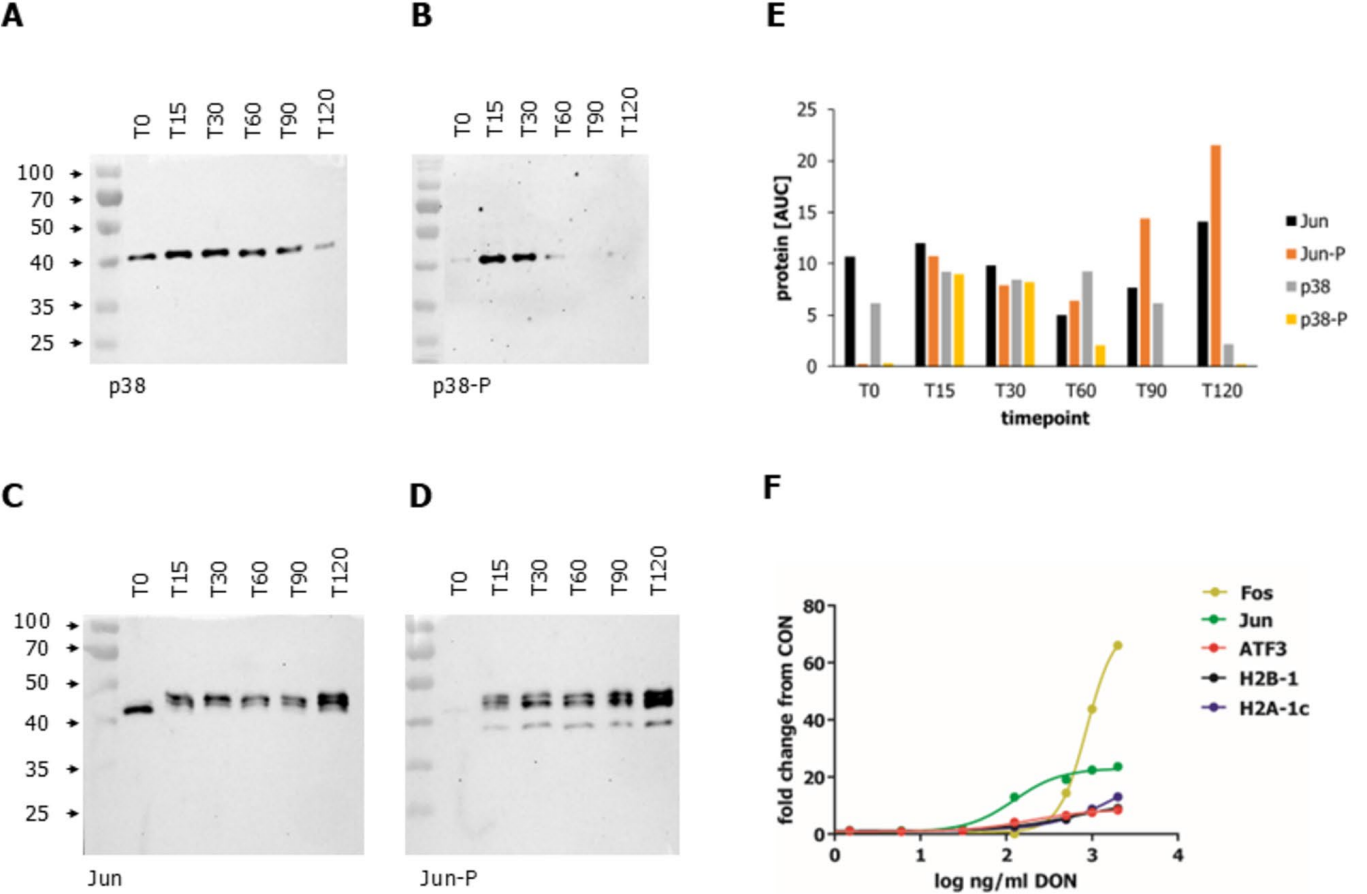
Western blot analysis of p38 (**Panel A**), phospho-p38 (p38-P; **Panel B**), Jun (**Panel C**) and phospho-Jun (Jun-P; **Panel D**) expression after exposure to 1000 ng/ml DON for different time periods (15 min., 30 min., 60 min., 90 min., 120 min.). **Panel E:** Western blot signals quantified from TIF files using ImageJ shown as area under the curve [AUC]. **Panel F:** Expression of the DON responsive genes Fos, Jun, ATF3, H2B-1 and H2A-1c in response to a range of DON concentrations (0.1 ng/ml to 2000 ng/ml). Expression of the genes was correlated with the control gene β-actin and is shown as fold change from gene expression in control cells treated with vehicle

We next analysed whether the exposure to DON also induces the expression of Jun protein. A recent proteomics analysis of A431 cells exposed to different concentrations of DON showed an increase in Jun protein abundance after a 24 h exposure to a concentration of 10uM DON (equivalent to 3000 ng/ml; i.e. 6 times higher than the concentration used in our analysis) (Del Favero et al. 2021). A Western blot analysis of V79 cells exposed to DON over 15, 30, 60, 90 and 120 min demonstrates an increase in total Jun protein abundance after 120 min of DON exposure (Fig. 5c, e). However, after only 15 min of exposure there is a strong increase in Jun phosphorylation (as evidenced by Western blot analysis using a phospho-Jun specific antibody; Fig. 5d, e). This confirms that the modification of the Jun protein into its active phosphorylated form (Minden et al. 1994) occurs very rapidly in response to DON exposure, whereas the increase in overall Jun protein expression is a slower process and can only be detected after 120 min of DON exposure. Phosphorylation of Jun protein occurs with the same timing as the phosphorylation of the MAP kinase protein p38 (Fig. 5a, b).

### Dose response to DON exposure

As a next step the dose response of the immediate early genes c-Fos, c-Jun and ATF3 and the histone genes H2A-1c and H2B-1 to DON exposure was assessed using a range of DON concentrations (between final concentrations of 0.03 ng/ml and 2000 ng/ml) for 90 min. The results demonstrate that the expression of all genes increases in response to DON treatment (Fig. 5f). The Jun gene responds most sensitively to DON exposure with a half-maximum activation (EC50) at a concentration of 124 ng/ml (supplementary Table 11). This is almost identical to the IC50 concentration for the Cell Titre Blue assay for cell viability (119 ng/ml; supplementary Table 2). The limit of detection (LOD) for all 3 immediate early response genes (Jun, Fos, ATF3) is around 30 ng/ml (supplementary Table 11). The IC50 value for the Fos gene is fourfold higher (863 ng/ml) than the IC50 for expression of the Jun gene. However, this is the consequence of the fact that the Fos gene is induced with a much higher amplitude (up to 70-fold) compared to the Jun and ATF3 genes (20-fold and tenfold induction of gene expression, respectively) (supplementary Table 11). These data suggest that the activation of immediate early genes can acts as a sensitive marker of trichothecene mycotoxin exposure.

## Discussion

Using RNA sequencing we have thoroughly analysed the response of mammalian cells to the trichothecene mycotoxins deoxynivalenol (DON) and HT2 through the first 120 min of toxin exposure. The data demonstrate that immediate early genes activated by the MAP kinase signalling pathway are the main targets of the cells’ response to trichothecene toxin exposure. While the role of immediate early gene expression has been identified previously (Pestka 2010), our datasets extend these findings and confirm that immediate early genes indeed constitute the main transcriptional response to trichothecene toxin exposure in mammalian cells. Most previous transcriptome analyses carried out in cells exposed to deoxynivalenol were carried out after a 24 h exposure (Hochstenbach et al. 2010; Pierron et al. 2016b; Alassane-Kpembi et al. 2017; Kalt et al. 2019; He et al. 2021; Tremblay-Franco et al. 2021). By this timepoint, significant transcriptional responses are still observed, however, the transcriptional responses with the highest amplitude in gene induction are no longer detectable.

The MAP kinase signalling pathway activates immediate early genes in different cellular scenarios. The best characterised pathways are [a] the cell stress response, which leads to the activation of apoptotic responses and  [b] the serum response which leads to cell proliferation (Zarubin and Han 2005; Faust et al. 2012; Yue and López 2020). The cellular environment and other signalling pathways determine whether p38 MAP kinase activation and its downstream sequelae lead to cell death or cell proliferation. Our results show surprisingly, that while mycotoxin exposure is clearly an inducer of cell stress and apoptosis gene pathways (activation of programmed cell death pathways GO:0012501 and GO:0043068 is observed at timepoints T60 and T90; supplementary Table 8), markers of cell proliferation (like c-myc) were upregulated too. We have previously observed that pro- and anti-apoptotic genes are both co-ordinately upregulated in response to endoplasmic reticulum stress in the mammary gland of α-casein deficient mice (Kolb et al. 2011). This suggests that cells respond to stressors by simultaneously accessing transcriptional programmes promoting cell death and cell survival.

DON and HT2 toxin exposure leads to a coordinated increase in the abundance of histone mRNA. Histone RNAs are upregulated by a post translational mechanism to ensure that sufficient amount of histone proteins are synthesized if a cell goes into the synthesis phase of the cell cycle (Rattray and Müller 2012). Histone gene expression is typically induced if cells enter the cell cycle in response to a proliferative stimulus. Analysis of publicly available datasets of other inducers of ribotoxic stress demonstrates that these agents also lead to an increase in histone gene expression. This includes anisomycin which binds to the same pocket on the large ribosomal subunit as HT2 and DON (Garreau De Loubresse et al. 2014; Sung et al. 2023). Furthermore, the Legionella protein SidI (a ribotoxic stressor which operates by inhibiting translation by acting as a non-functional t-RNA mimic) also leads to an upregulation of histone mRNA abundance (Subramanian et al. 2023). The biggest transient increase of histone expression is found for H1 histones and H2B histones, whereas H2A histones are only marginally upregulated overall. The transcriptional changes typically only affect a select subset of histone genes (e.g., 2 genes of 11 for histone H1, 9 of 35 genes for histone H2A). The upregulated histone genes are mostly canonical histones associated with cell proliferation, rather than non-canonical histones which are typically activated under stress conditions (Halliwell et al. 2020; Amatori et al. 2021). This suggests that the effect of ribotoxic trichothecene toxins elicit transcriptional responses in histone genes which resemble proliferative signals rather than cell stress signals.

Pathway analysis demonstrated that numerous chemicals in the LINCS 1000 database also activate the immediate early gene response (including the phosphatase inhibitor okadaic acid or the ATPase inhibitor digitoxigenin). However, after 24 h of exposure to trichothecene mycotoxins most of the gene expression changes detected are shared with other translation inhibitors like Emetine (a plant toxin), Narciclasine, Cephaeline (two plant alkaloid toxins) or Bruceantin (a plant triterpenoid).

Immediate early gene transcription occurs rapidly, apparently in the absence of the need for de novo protein translation (Bahrami and Drabløs 2016). Trichothecene toxins reduce the efficiency of protein translation. Activation of immediate early genes may therefore escape the effects of impaired translation. The activation of the Fos and Jun genes induced by trichothecene toxins and the growth promoter PMA are similar. This suggests that that the transcriptional programmes induced by different environmental stimuli can overlap significantly. This interpretation is also supported by pathway analysis using the G-Profiler and EnrichR platforms. E.g., the genes activated by DON exposure show significant similarity to genes activated by the inactivation of the cell cycle regulator CHEK1 or the pro-apoptotic gene PDCD10. In other words, removing a block in cell cycle progression (promoting cell cycle progression) activates a similar subset of genes which are activated by DON exposure. However, toxin exposure will eventually impair cell viability whereas PMA will promote cell growth.

The activation of immediate early genes can also be detected in the B-cell precursor cell line K562 in which transcriptional changes in response to PMA treatment were analysed over time (Limb et al. 2009). Intriguingly while there is a rapid activation of some histone genes during the first 30 min of PMA treatment, the increases are only up to 1.5-fold. This is consistent with our observations in V79 cells treated with PMA (data not shown). This contrasts with the effect of other cell stressors. E.g., we have detected that histone gene mRNA abundance is reduced in response to treatment of human Caco_2_ intestinal cells with the oxidative stress inducer sulforaphane (acting via the transcription factor Nrf2) or flavonoid inducers of the Ahr (aryl-hydrocarbon receptor) signalling pathway (Harbottle and Kolb, unpublished).

Overall our data show that [1] immediate early gene activation is a rapid but transient response to DON or HT2 exposure, [2] this response is shared with numerous unrelated stimuli (including inducers of cell proliferation and other toxins), [3] rapid responses to DON exposure activate histone gene transcription despite DON exposure not promoting cell proliferation, and [4] long term transcriptome changes in response to DON specifically overlap with the effects of other inhibitors of translation. Finally, our data also show that the response to DON exposure leads to a rapid and sensitive activation of specific gene sets which can be exploited for the development of novel sensitive bioassays for toxin exposure.

## Supplementary Information

Below is the link to the electronic supplementary material.

Supplementary file1 (PDF 668 KB)

## Data Availability

The RNA sequencing data will be deposited to the NCBI GEO database once that manuscript is accepted for publication.

## Abbreviations

DON: Deoxynivalenol
EFSA: European food standards agency
EC50: Half maximal effective concentration
FPKM: Fragments per kilobase of transcript per million mapped reads
FSA: Food standards agency
FDA: Food and drug administration
IC50: Half maximal inhibitory concentration
LC-MS: Liquid chromatography-mass spectrometry
LOD: Limit of detection
PMA: Phorbol 12-myristate 13-acetate

## References

[CR1] Alassane-Kpembi I, Gerez JR, Cossalter A-MM et al (2017) Intestinal toxicity of the type B trichothecene mycotoxin fusarenon-X: whole transcriptome profiling reveals new signaling pathways. Sci Rep 7:7530. 10.1038/s41598-017-07155-2 28790326 10.1038/s41598-017-07155-2 PMC5548841

[CR2] Alvito P, Assunção RM, Bajard L et al (2022) Current advances, research needs and gaps in Mycotoxins biomonitoring under the HBM4EU—lessons learned and future trends. Toxins (Basel) 14:826. 10.3390/toxins14120826 36548723 10.3390/toxins14120826 PMC9783896

[CR3] Amatori S, Tavolaro S, Gambardella S, Fanelli M (2021) The dark side of histones: genomic organization and role of oncohistones in cancer. Clin Epigenetics 13:71. 10.1186/s13148-021-01057-x 33827674 10.1186/s13148-021-01057-x PMC8025322

[CR4] Bae HK, Pestka JJ (2008) Deoxynivalenol induces p38 interaction with the ribosome in monocytes and macrophages. Toxicol Sci 105:59–66. 10.1093/toxsci/kfn102 18502741 10.1093/toxsci/kfn102 PMC6592419

[CR5] Bahrami S, Drabløs F (2016) Gene regulation in the immediate-early response process. Adv Biol Regul 62:37–49. 10.1016/J.JBIOR.2016.05.001 27220739 10.1016/j.jbior.2016.05.001

[CR6] Cheli F, Giromini C, Baldi A (2015) Mycotoxin mechanisms of action and health impact: In vitro or in vivo tests, that is the question. World Mycotoxin J. 10.3920/WMJ2014.1864

[CR7] Daud N, Currie V, Duncan G et al (2020) Prevalent human gut bacteria hydrolyse and metabolise important food-derived mycotoxins and masked mycotoxins. Toxins (Basel) 12:654. 10.3390/toxins12100654 33066173 10.3390/toxins12100654 PMC7601956

[CR8] Del Favero G, Janker L, Neuditschko B et al (2021) Exploring the dermotoxicity of the mycotoxin deoxynivalenol: combined morphologic and proteomic profiling of human epidermal cells reveals alteration of lipid biosynthesis machinery and membrane structural integrity relevant for skin barrier function. Arch Toxicol 95:2201–2221. 10.1007/s00204-021-03042-y 33890134 10.1007/s00204-021-03042-y PMC8166681

[CR9] Doehmer J (1993) V79 Chinese hamster cells genetically engineered for cytochrome P450 and their use in mutagenicity and metabolism studies. Toxicology 82:105–118. 10.1016/0300-483X(93)90063-X 8236270 10.1016/0300-483x(93)90063-x

[CR10] Evangelista JE, Xie Z, Marino GB et al (2023) Enrichr-KG: bridging enrichment analysis across multiple libraries. Nucleic Acids Res 51:W168–W179. 10.1093/NAR/GKAD393 37166973 10.1093/nar/gkad393 PMC10320098

[CR11] Faust D, Schmitt C, Oesch F et al (2012) Differential p38-dependent signalling in response to cellular stress and mitogenic stimulation in fibroblasts. Cell Commun Signal 10:1–13. 10.1186/1478-811X-10-6 22404972 10.1186/1478-811X-10-6 PMC3352310

[CR12] Garreau De Loubresse N, Prokhorova I, Holtkamp W et al (2014) Structural basis for the inhibition of the eukaryotic ribosome. Nature 513:517–522. 10.1038/NATURE13737 25209664 10.1038/nature13737

[CR13] Gratz S (2017) Do plant-bound masked mycotoxins contribute to toxicity? Toxins (Basel) 9:85. 10.3390/toxins9030085 28264486 10.3390/toxins9030085 PMC5371840

[CR14] Gratz SW, Duncan G, Richardson AJ (2013) The human fecal microbiota metabolizes deoxynivalenol and deoxynivalenol-3-glucoside and may be responsible for urinary deepoxy-deoxynivalenol. Appl Environ Microbiol 79:1821–1825. 10.1128/AEM.02987-12 23315729 10.1128/AEM.02987-12 PMC3592229

[CR15] Gutiérrez S, McCormick SP, Cardoza RE et al (2021) Distribution, function, and evolution of a gene essential for Trichothecene toxin biosynthesis in trichoderma. Front Microbiol 12:791641. 10.3389/FMICB.2021.791641/BIBTEX 34925301 10.3389/fmicb.2021.791641 PMC8675399

[CR16] Halliwell JA, Frith TJR, Laing O et al (2020) Nucleosides rescue replication-mediated genome instability of human pluripotent stem cells. Stem Cell Reports 14:1009. 10.1016/J.STEMCR.2020.04.004 32413278 10.1016/j.stemcr.2020.04.004 PMC7355123

[CR17] He K, Zhou HR, Pestka JJ (2012) Targets and intracellular signaling mechanisms for deoxynivalenol-induced ribosomal RNA cleavage. Toxicol Sci 127:382. 10.1093/TOXSCI/KFS134 22491426 10.1093/toxsci/kfs134 PMC3355321

[CR18] He Y, Yin X, Dong J et al (2021) Transcriptome analysis of Caco-2 cells upon the exposure of mycotoxin deoxynivalenol and its acetylated derivatives. Toxins (Basel) 13:167. 10.3390/toxins13020167 33671637 10.3390/toxins13020167 PMC7927021

[CR19] Hochstenbach K, van Leeuwen DM, Gmuender H et al (2010) Transcriptomic profile indicative of immunotoxic exposure: in vitro studies in peripheral blood mononuclear cells. Toxicol Sci 118:19–30. 10.1093/toxsci/kfq239 20702593 10.1093/toxsci/kfq239

[CR20] Hooft JM, Bureau DP (2021) Deoxynivalenol: mechanisms of action and its effects on various terrestrial and aquatic species. Food Chem Toxicol 157:112616. 10.1016/j.fct.2021.112616 34662691 10.1016/j.fct.2021.112616

[CR21] Jia H, Wu WD, Lu X et al (2017) Role of glucagon-like Peptide-1 and gastric inhibitory peptide in anorexia induction following oral exposure to the trichothecene mycotoxin deoxynivalenol (Vomitoxin). Toxicol Sci 159:16–24. 10.1093/TOXSCI/KFX112 28633506 10.1093/toxsci/kfx112

[CR22] Juan-García A, Juan C, Tolosa J, Ruiz MJ (2019) Effects of deoxynivalenol, 3-acetyl-deoxynivalenol and 15-acetyl-deoxynivalenol on parameters associated with oxidative stress in HepG2 cells. Mycotoxin Res 35:197–205. 10.1007/s12550-019-00344-0 30806951 10.1007/s12550-019-00344-0

[CR23] Kalt W, Cassidy A, Howard LR et al (2019) Recent research on the health benefits of blueberries and their anthocyanins. Adv Nutr 11:224–236. 10.1093/advances/nmz065 10.1093/advances/nmz065 PMC7442370 31329250

[CR24] Kimura M, Kaneko I, Komiyama M et al (1998) Trichothecene 3-O-acetyltransferase protects both the producing organism and transformed yeast from related mycotoxins: cloning and characterization of Tri101. J Biol Chem 273:1654–1661. 10.1074/JBC.273.3.1654 9430709 10.1074/jbc.273.3.1654

[CR25] Kolb AF, Huber RC, Lillico SG et al (2011) Milk lacking α-casein leads to permanent reduction in body size in mice. PLoS ONE 6:e21775. 10.1371/journal.pone.0021775 21789179 10.1371/journal.pone.0021775 PMC3138747

[CR26] Kuleshov MV, Jones MR, Rouillard AD et al (2016) Enrichr: a comprehensive gene set enrichment analysis web server 2016 update. Nucleic Acids Res 44:W90–W97. 10.1093/nar/gkw377 27141961 10.1093/nar/gkw377 PMC4987924

[CR27] Limb JK, Yoon S, Lee KE et al (2009) Regulation of megakaryocytic differentiation of K562 cells by FosB, a member of the Fos family of AP-1 transcription factors. Cell Mol Life Sci 66:1962–1973. 10.1007/s00018-009-8775-5 19381435 10.1007/s00018-009-8775-5 PMC11115838

[CR28] Liu Y, Glatt H (2010) Human cytochrome P450 2E1 and sulfotransferase 1A1 coexpressed in Chinese hamster V79 cells enhance spontaneous mutagenesis. Environ Mol Mutagen 51:23–30. 10.1002/EM.20503 19484729 10.1002/em.20503

[CR29] Lyagin I, Efremenko E (2019) Enzymes for detoxification of various mycotoxins: origins and mechanisms of catalytic action. Molecules. 10.3390/molecules24132362 31247992 10.3390/molecules24132362 PMC6651818

[CR30] Minden A, Lin A, Smeal T et al (1994) c-Jun N-terminal phosphorylation correlates with activation of the JNK subgroup but not the ERK subgroup of mitogen-activated protein kinases. Mol Cell Biol 14:6683–6688. 10.1128/mcb.14.10.6683-6688.1994 7935387 10.1128/mcb.14.10.6683-6688.1994 PMC359198

[CR31] Moon Y, Pestka JJ (2003) Deoxynivalenol-induced mitogen-activated protein kinase phosphorylation and IL-6 expression in mice suppressed by fish oil. J Nutr Biochem 14:717–726. 10.1016/j.jnutbio.2003.08.009 14690764 10.1016/j.jnutbio.2003.08.009

[CR32] Nesic K, Ivanovic S, Nesic V (2014) Fusarial toxins: secondary metabolites of *Fusarium* fungi. Rev Environ Contam Toxicol 228:101–120. 10.1007/978-3-319-01619-1_5 24162094 10.1007/978-3-319-01619-1_5

[CR33] Nolan P, Auer S, Spehar A et al (2019) Current trends in rapid tests for mycotoxins. Food Addit Contam Part A Chem Anal Control Expo Risk Assess 36:800–814. 10.1080/19440049.2019.1595171 30943116 10.1080/19440049.2019.1595171

[CR34] Pestka JJ (2010) Deoxynivalenol: mechanisms of action, human exposure, and toxicological relevance. Arch Toxicol 84:663–679. 10.1007/s00204-010-0579-8 20798930 10.1007/s00204-010-0579-8

[CR35] Pestka JJ, Smolinski AT (2005) Deoxynivalenol: toxicology and potential effects on humans. J Toxicol Environ Health B Crit Rev 8:39–69. 10.1080/10937400590889458 15762554 10.1080/10937400590889458

[CR36] Pierron A, Mimoun S, Murate LS et al (2016a) Microbial biotransformation of DON: molecular basis for reduced toxicity. Sci Rep 6:29105. 10.1038/srep29105 27381510 10.1038/srep29105 PMC4933977

[CR37] Pierron A, Mimoun S, Murate LS et al (2016b) Intestinal toxicity of the masked mycotoxin deoxynivalenol-3-β-d-glucoside. Arch Toxicol 90:2037–2046. 10.1007/s00204-015-1592-8 26404761 10.1007/s00204-015-1592-8

[CR38] Polak-śliwińska M, Paszczyk B (2021) Trichothecenes in food and feed, relevance to human and animal health and methods of detection: a systematic review. Molecules 26:454. 10.3390/molecules26020454 33467103 10.3390/molecules26020454 PMC7830705

[CR39] Rattray AMJ, Müller B (2012) The control of histone gene expression. Biochem Soc Trans 40:880–885. 10.1042/BST20120065 22817752 10.1042/BST20120065

[CR40] Reimand J, Isserlin R, Voisin V et al (2019) Pathway enrichment analysis and visualization of omics data using g:Profiler, GSEA, Cytoscape and EnrichmentMap. Nat Protoc 14:482–517. 10.1038/s41596-018-0103-9 30664679 10.1038/s41596-018-0103-9 PMC6607905

[CR41] Shi Y, Porter K, Parameswaran N et al (2009) Role of GRP78/BiP degradation and ER stress in deoxynivalenol-induced interleukin-6 upregulation in the macrophage. Toxicol Sci 109:247–255. 10.1093/TOXSCI/KFP060 19336499 10.1093/toxsci/kfp060 PMC2721661

[CR42] Shifrin VI, Anderson P (1999) Trichothecene mycotoxins trigger a ribotoxic stress response that activates c-jun n-terminal kinase and p38 mitogen-activated protein kinase and induces apoptosis. J Biol Chem 274:13985–13992. 10.1074/jbc.274.20.13985 10318810 10.1074/jbc.274.20.13985

[CR43] Subramanian A, Narayan R, Corsello SM et al (2017) A next generation connectivity map: L1000 platform and the first 1,000,000 profiles. Cell 171:1437-1452.e17. 10.1016/j.cell.2017.10.049 29195078 10.1016/j.cell.2017.10.049 PMC5990023

[CR44] Subramanian A, Wang L, Moss T et al (2023) A *Legionella* toxin exhibits tRNA mimicry and glycosyl transferase activity to target the translation machinery and trigger a ribotoxic stress response. Nat Cell Biol 25:1600–1615. 10.1038/s41556-023-01248-z 37857833 10.1038/s41556-023-01248-z PMC11005034

[CR45] Sugiyama K, Muroi M, Kinoshita M et al (2016) NF-κB activation via MyD88-dependent Toll-like receptor signaling is inhibited by trichothecene mycotoxin deoxynivalenol. J Toxicol Sci 41:273–279. 10.2131/jts.41.273 26961612 10.2131/jts.41.273

[CR46] Sung HM, Schott J, Boss P, Lehmann JA, Hardt MR, Lindner D, Messens J, Bogeski I, Ohler U, Stoecklin G (2023) Stress-induced nuclear speckle reorganization is linked to activation of immediate early gene splicing. J Cell Biol 222(12):e202111151 37956386 10.1083/jcb.202111151 PMC10641589

[CR47] Szymanowska M, Hendry KAK, Robinson C, Kolb AF (2009) EMMPRIN (basigin/CD147) expression is not correlated with MMP activity during adult mouse mammary gland development. J Cell Biochem 106:52–62. 10.1002/jcb.21975 19003972 10.1002/jcb.21975

[CR48] Tolosa L, Donato MT, Pérez-Cataldo G et al (2012) Upgrading cytochrome P450 activity in HepG2 cells co-transfected with adenoviral vectors for drug hepatotoxicity assessment. Toxicol Vitro 26:1272–1277. 10.1016/j.tiv.2011.11.008 10.1016/j.tiv.2011.11.008 22138474

[CR49] Tremblay-Franco M, Canlet C, Pinton P, Lippi Y, Gautier R, Naylies C, Neves M, Oswald IP, Debrauwer L, Alassane-Kpembi I (2021) Statistical integration of ‘omics data increases biological knowledge extracted from metabolomics data: application to intestinal exposure to the mycotoxin deoxynivalenol. Metabolites 11(6):407 34205708 10.3390/metabo11060407 PMC8233929

[CR50] Vind AC, Genzor AV, Bekker-Jensen S (2020a) Ribosomal stress-surveillance: three pathways is a magic number. Nucleic Acids Res 48:10648–10661. 10.1093/nar/gkaa757 32941609 10.1093/nar/gkaa757 PMC7641731

[CR51] Vind AC, Snieckute G, Blasius M et al (2020b) ZAKα recognizes stalled ribosomes through partially redundant sensor domains. Mol Cell 78:700-713.e7. 10.1016/j.molcel.2020.03.021 32289254 10.1016/j.molcel.2020.03.021

[CR52] Yang C, Song G, Lim W (2020) Effects of mycotoxin-contaminated feed on farm animals. J Hazardous Mater 5(389):122087 10.1016/j.jhazmat.2020.122087 32004836

[CR53] Yue J, López JM (2020) Understanding MAPK signaling pathways in apoptosis. Int J Mol Sci 21:2346. 10.3390/IJMS21072346 32231094 10.3390/ijms21072346 PMC7177758

[CR54] Yue J, Guo D, Gao X et al (2021) Deoxynivalenol (Vomitoxin)-induced anorexia is induced by the release of intestinal hormones in mice. Toxins (Basel). 10.3390/TOXINS13080512 34437383 10.3390/toxins13080512 PMC8402572

[CR55] Zarubin T, Han J (2005) Activation and signaling of the p38 MAP kinase pathway. Cell Res 15:11–18. 10.1038/sj.cr.7290257 15686620 10.1038/sj.cr.7290257

[CR56] Zhou HR, Pestka JJ (2015) Deoxynivalenol (vomitoxin)-induced cholecystokinin and glucagon-like peptide-1 release in the STC-1 enteroendocrine cell model is mediated by calcium- sensing receptor and transient receptor potential ankyrin-1 channel. Toxicol Sci 145:407–417. 10.1093/toxsci/kfv061 25787141 10.1093/toxsci/kfv061 PMC4542861

