# Supplementary material for: Trichothecene mycotoxin-induced ribotoxic stress activates histone gene transcription
Source: Arch Toxicol. 2026 Mar 31;100(7):3185–97. doi: 10.1007/s00204-026-04375-2 (PMC13309516; doi:10.1007/s00204-026-04375-2)
Supplement: Supplementary file 1 — Supplementary file1 (PDF 668 KB) [file 204_2026_4375_MOESM1_ESM.pdf]

Trichothecene mycotoxin induced ribotoxic stress activates histone gene transcription

Andreas F. Kolb[1,2], Vanina Popova[1], Linda Petrie[1]

[1]Nutrition, Obesity and Disease Research Theme, Rowett Institute, University of Aberdeen, UK,

Supplementary materials

Supplementary tables

name	sequence	ann. temp.	amplicon size
bact3	5' GAYGAGGCYCAGAGCAAGAGAG 3'	60°C	387bp
bact4	5' GTCCCGGCCAGCCAGGTCCAG 3'		
chJun-F1	5' GACAGTTCCGTGACGACTGG 3'	61°C	122bp
chJun-R1	5' GCTTTGAAAAGTCGCGGTCA 3'		
chFos3	5' CCTTCTCACTGCCTCTTCTC 3'	52°C	181bp
chFos4	5' GAACCGGATAGGTCCATGTC 3'		
chATF3-F2	5' AACCCTGAAGCCGTTGGAAA 3'	55°C	157bp
chATF3-R2	5' TAGTGAGACAAGGGACGCTC 3'		
chEgr1-F	5' CGGGGAGTCGCGAGAGAT 3'	60°C	377bp
chEgr1-R	5' GGGTGAGTGAGGAAAGGAGC 3'		
chH1.2-F1	5' GCGCTAGTCAGTGTTGGACT 3'	60°C	334bp
chH1.2-R1	5' GTTTGACCCGCCACTCCCTA 3'		
chH2A-F1	5' GAGAGCCACCATAAGGCCAA 3'	60°C	166bp
chH2A-R1	5' CACGCGACAACCTACACTTC 3'		
chH2B-F1	5' TACAACAAGCGCTCGACCAT 3'	60°C	149bp
chH2B-R1	5' CTCGTGGGACGTCCTTTTCA 3'		

Supplementary Table 1: Oligonucleotide primer pairs used for quantitative PCR experiments in this study. The chH1.2-F1 and R1 primer pair is specific to Cricetulus griseus histone H1.2 (LOC100752904). The chH2A-F1 and R1 primer pair is specific to Cricetulus griseus histone H2A type 2-A (LOC100758065). The chH2B-F1 and R1 primer pair is specific to Cricetulus griseus histone H2B type 1-C/E/F/G/I (LOC100752010), transcript variant X1.

toxin	cell confluence	IC25	IC50	IC75
DON	30%	67	119	211
	70%	196	318	516
HT2	30%	4	9	19
	70%	11	25	57
ANI	30%	5	9	16
	70%	16	40	103

Supplementary table 2: IC25, IC50, and IC75 values (in ng/ml) for the viability of V79 Chinese Hamster lung fibroblasts in response to treatment with deoxynivalenol (DON), HT2 or anisomycin (ANI).

gene	av T0 FPKM	gene	av T15 FPKM	gene	av T30 FPKM	gene	av T60 FPKM	gene	av T90 FPKM	gene	av T120 FPKM	gene	av T24 FPKM
COX1	10785.30	COX1	11955.11	COX1	10355.17	COX1	10356.71	COX1	9758.27	COX1	8408.15	COX1	7121.26
COX3	6752.57	COX3	7580.94	COX3	6633.14	COX3	6454.57	COX3	6298.09	COX3	5517.68	ATP6	6626.97
Eef1a1	5408.17	ATP6	7250.20	Eef1a1	5459.78	Eef1a1	5649.82	Eef1a1	5541.83	Eef1a1	5309.37	Eef1a1	6085.85
ATP6	5342.85	ND2	6166.86	ATP6	5335.43	ATP6	5305.27	ATP6	5219.48	ATP6	4624.40	ATP8	5908.97
ND2	5184.88	ATP8	6094.05	ND2	4613.65	ATP8	4669.85	ATP8	4496.97	ATP8	4127.05	COX3	5281.09
ATP8	4681.76	Eef1a1	5382.83	ATP8	4571.67	ND2	4576.93	ND2	4325.27	ND2	3851.81	ND2	4196.85
COX2	3462.06	COX2	4194.10	COX2	3367.00	COX2	3188.87	COX2	3154.71	COX2	2739.50	**Rpl23a**	4061.19
Rps2	2997.18	ND4	4087.90	ND4	3050.35	Rps2	2853.56	CYTB	2803.59	Rps2	2607.02	CYTB	3491.35
ND4	2789.92	ND1	3286.76	Rps2	2955.08	CYTB	2817.91	Rps2	2785.52	CYTB	2529.97	Rps2	3126.44
**Rpl23a**	2737.17	CYTB	3181.92	**Rpl23a**	2725.16	**Rpl23a**	2788.15	ND4	2743.61	**Rpl23a**	2517.33	COX2	2688.43
Ptma	2629.59	Rps2	2843.07	CYTB	2706.62	ND4	2663.75	**Rpl23a**	2698.05	LOC100764022	2357.83	ND1	2561.49
CYTB	2574.42	ND4L	2780.74	Ptma	2524.54	Ptma	2517.91	ND1	2471.76	ND4	2353.29	ND4	2316.73
ND1	2553.62	**Rpl23a**	2547.65	ND1	2464.29	ND1	2453.57	Ptma	2265.91	Actg1	2238.01	Ubb	2292.87
Gapdh	2165.41	Ptma	2542.52	Hspa8	2222.26	LOC100761791	1966.70	ND4L	1935.64	ND1	2185.36	Rpl6	2114.86
LOC113830659	1952.44	Hspa8	2186.94	ND4L	2158.37	Gapdh	1912.56	LOC100764022	1875.86	Ptma	1982.35	Gapdh	2089.90
Rpsa	1943.88	Gapdh	1967.56	Gapdh	2008.32	Rpsa	1908.07	LOC100761791	1859.87	Actb	1844.89	LOC100761791	1961.97
ND4L	1910.37	LOC100761791	1867.48	Rpsa	1906.33	ND4L	1897.74	Gapdh	1859.77	Thbs1	1815.86	Eif4a1	1930.41
Hspa8	1889.26	Rpsa	1857.27	LOC113830659	1850.53	Actb	1878.27	Rpsa	1859.44	Gapdh	1763.31	Rplp0	1859.10
LOC100754792	1843.84	ND6	1836.67	LOC100754792	1818.05	LOC100764022	1809.24	Actg1	1787.11	Ubb	1752.95	Anxa2	1735.27
LOC100761791	1767.85	ND5	1829.64	LOC100761791	1806.26	LOC100754792	1790.15	Actb	1732.28	LOC100761791	1746.90	Rpsa	1729.74
Actb	1754.08	LOC113830659	1810.06	Rpl21	1727.22	LOC113830659	1746.71	LOC100754792	1702.62	Rpsa	1728.71	Rack1	1705.71
Rpl21	1728.61	LOC100754792	1807.43	Actb	1682.68	Actg1	1738.26	LOC113830659	1682.30	ND4L	1683.30	ND4L	1675.77
Rplp0	1710.73	Actb	1713.69	Rplp0	1676.11	Hspa8	1728.82	Rplp0	1675.36	LOC100754792	1614.15	Rps27a	1673.34
Pfn1	1651.67	Fth1	1655.96	Ppia	1663.16	Rpl21	1694.10	Rpl21	1650.45	Tpt1	1585.17	Rps7	1662.72
Ppia	1648.58	Rpl21	1579.07	Pfn1	1639.45	Rplp0	1674.57	Tpt1	1639.31	Rplp0	1580.24	Ptma	1642.17
Fth1	1633.94	Rps3a	1577.43	Fth1	1606.66	Tpt1	1635.71	Ppia	1612.88	LOC113830659	1571.09	Rps3a	1622.98
Rps3a	1586.18	Rplp0	1571.67	Tpt1	1588.17	Ppia	1625.39	Ubb	1538.47	Rpl21	1535.83	LOC113830659	1583.16
Tpt1	1557.94	Ppia	1545.04	LOC100764022	1571.00	Rps3a	1571.92	Rps3a	1516.65	Ppia	1489.38	Tpt1	1538.41
Rps4x	1556.67	Rps4x	1542.57	Rps4x	1568.60	Rps4x	1513.71	Rps4x	1494.11	Rps3a	1409.19	Rps8	1529.27
Actg1	1549.17	Pfn1	1538.28	Rps3a	1562.16	Pfn1	1513.18	Rpl6	1464.68	Rpl6	1395.41	Rps4x	1520.19
Rpl6	1542.06	LOC100764022	1522.54	Actg1	1559.34	Rpl6	1510.22	Hspa8	1447.89	Rps4x	1395.31	Rpl21	1499.94
Tuba1b	1541.68	Rpl6	1503.96	Rpl6	1540.74	Fth1	1465.20	Rack1	1433.05	Rack1	1388.86	Ppia	1490.02
LOC100764022	1541.15	Tpt1	1499.94	Gstp1	1496.86	Rack1	1459.01	Rps27a	1427.29	**Rpl3**	1353.29	Rpl13a	1477.63
Rack1	1536.70	Tuba1b	1480.02	Rack1	1491.74	Rps27a	1444.94	Rps24	1399.35	Pfn1	1345.37	Rps14	1437.74
Rps27a	1458.14	Actg1	1450.88	Tuba1b	1491.70	Rps24	1425.02	Pfn1	1394.20	Rps27a	1282.83	Rpl4	1411.88
Rps24	1420.61	Rack1	1421.99	Rps24	1450.39	Tuba1b	1416.57	Fth1	1379.51	Fth1	1269.84	LOC100754792	1404.95
Gstp1	1389.03	Gstp1	1384.45	Rps27a	1445.90	Ubb	1405.48	Tuba1b	1350.18	Tuba1b	1269.51	**Rpl11**	1394.35
Rps7	1352.10	Rps27a	1376.29	Rps14	1376.00	Rps14	1340.75	ND6	1347.90	Rps24	1268.80	Eef1g	1383.01
Rps14	1347.04	Rps24	1360.04	ND6	1356.05	ND6	1336.49	**Rpl3**	1324.95	Eif4a1	1262.81	LOC100767070	1376.33
ND6	1316.00	Prdx1	1294.75	Rps7	1342.93	Rps7	1335.46	Rps14	1307.67	ND6	1245.14	Rps24	1337.48
Pkm	1296.30	Rps7	1290.95	Prdx1	1322.29	**Rpl3**	1306.90	Thbs1	1302.74	Rps7	1217.49	--	1333.39
ND5	1289.94	Rps14	1288.44	ND5	1312.42	Gstp1	1300.06	Rps7	1285.94	Rps14	1213.15	Rpl7a	1326.10
LOC100759352	1279.35	Rps8	1227.82	LOC100759352	1297.04	LOC100759352	1295.07	ND5	1271.16	Eef2	1173.25	Rpl7	1308.80
Eef2	1272.98	Set	1222.89	Rps8	1271.15	ND5	1233.24	Gstp1	1267.07	LOC100759352	1166.49	Rpl18	1303.69
Ybx1	1257.88	Ybx1	1222.30	Eef2	1241.98	Rps8	1231.91	LOC100759352	1238.30	Gstp1	1136.14	Pfn1	1293.21
Prdx1	1253.77	Eef2	1213.22	Rpl13a	1235.09	Rpl13a	1229.09	Eif4a1	1227.39	Rpl13a	1134.71	Set	1282.64
Rps8	1250.21	Rpl7	1197.91	Pkm	1227.06	Eef2	1217.35	Rps8	1198.45	Rps8	1122.11	Rps11	1280.81
Rpl13a	1230.03	Pkm	1195.93	Rpl7	1198.58	Eif4a1	1215.64	Rpl13a	1183.30	ND5	1121.16	Rps12	1234.48
Ldha	1210.85	Rpl13a	1145.37	Ubb	1190.70	Prdx1	1202.31	Prdx1	1180.66	Anxa2	1078.17	LOC100763972	1214.42
Set	1206.13	**Rpl3**	1139.86	**Rpl3**	1190.08	Rpl7	1187.35	Eef2	1172.92	Rps11	1077.44	Eef2	1204.02

Supplementary table 3. FPKM values (Fragments Per Kilobase of transcript per Million mapped reads) of the most highly expressed genes in V79 cells exposed to DON at the 7 timepoint of the experiment (T0, T15, T30, T60, T90, T120 and T24). Ribosomal genes are marked in brown. Mitochondrial genes belonging to 3 major gene families (COX: cytochrome oxidase; ATP: ATP synthase; ND: NADH dehydrogenase) are shown in yellow, blue and green, respectively. The typical reference genes β-actin and GAPDH are marked in grey.

name	fc T15/T0	gene	fc T30/T0	gene	fc T60/T0	gene	fc T90/T0	gene	fc T120/T0	gene	fc T24/T0
Gpr1	12.58	H1-6	114.57	H1-6	334.60	H1-6	188.89	Csf2	266.47	Gbx1	68.43
Dnah9	11.94	H2A-1	56.61	Fos	159.20	Csf2	160.60	H2B-1	149.95	LOC103162898	59.12
H2A-1b	9.71	Fos	39.18	H2B-1	144.04	H2B-1	154.12	H1-3	138.50	Fam131b	49.03
H4	9.21	H1-2	34.84	H1-3	93.02	H1-3	133.29	H1-2	137.34	Zfp420	32.59
Zscan18	8.73	H2A-1B	30.54	H1-2	87.37	H1-2	132.70	H2A-1b	92.87	Cts8	26.37
Mcidas	8.53	H2B-1B	28.69	H2A-1b	81.01	Fos	123.86	H2A-1b	69.90	TRIOBP	23.86
H1-2	8.46	H1-1	27.19	Smok2b	73.61	Smok2b	108.98	H2B-1	59.86	LOC118239606	21.40
PRC1	8.18	H4	24.09	H2B-1	70.30	H2A-1b	79.55	Fam131b	57.84	OR10T2	18.94
H3	7.55	H3-1	23.01	H2A-1b	69.40	VN1R4	77.09	H2A-1b	56.91	PRDM9	17.64
LOC100773314	6.74	H1-3	18.51	H2A-1b	64.49	H2A-1b	65.36	H2A-1b	51.79	H2A-2a	16.96
H3-1	6.55	H2A-1B	15.66	Rasgrp2	59.51	Rasgrp2	64.76	Smok2b	46.86	CMTM2	16.92
Fos	5.21	Egr1	15.00	H2A-1b	48.42	H2A-1b	50.46	Znrf4	44.00	Znf120	16.67
H3-1	4.96	LOC110308656	13.72	Egr1	46.26	H2A-1b	48.23	H1-1	42.19	H2B-1	16.44
H2B-1	4.73	Cfap44	13.32	Csf2	42.82	H2A-1b	48.10	H1-6	41.21	Shisa2	16.40
FHL1	3.98	H3-1	12.97	Jun	39.91	Jun	47.53	H2B-1	37.70	H1-2	16.02
Dnai2	3.88	H4	12.80	H1-1	35.88	H1-1	45.90	H2B-1k	36.62	H1-3	15.19
H4	3.74	H2B-1K	12.20	H2A-1b	35.81	Egr1	43.07	H2A-1b	36.58	Cmya5	15.03
Ypel2	3.72	H2A-1B	11.98	Magee1	35.60	H4	41.01	H3-3	36.39	TRIM15	14.79
Calcr	3.70	Jun	11.90	H2B-1k	35.47	H2bc12	37.79	H2B-1b	32.91	Kremen2	14.70
Kcnj12	3.54	H3	10.56	H2B-1	33.70	H2A-2a	36.90	H2B-1	32.16	H3-3	14.63
Nat8f1	3.52	H2B-1	10.21	H2B-1	32.19	H3-1	36.75	H2A-2a	32.12	PRKN	13.30
Marveld2	3.33	H3-1	9.99	Cysltr2	30.92	H2B-1	35.04	H2A-2a	31.35	Il17c	13.15
Gng13	3.27	H2A-1F	8.36	Hao1	30.25	H2A-2	34.44	H2B-1a	30.69	H2A-2a	12.99
H2A-1b	3.16	H2B-1	7.78	H4	28.98	H3-3	34.02	H2B-1	30.59	Pcdh15	12.67
H2A-1b	2.93	H2A-1B	7.76	H2B-1b	28.80	H2B-1b	33.71	H3-1	30.36	Galr2	12.33
MAPT	2.85	H2B-1	7.63	H2A-2a	28.51	H2B-1a	32.95	PRDM9	27.84	LOC118239975	12.20
ZNF146	2.72	H2B-1M	7.62	H3-3	28.12	H2B-1	31.24	Jun	26.39	Mapk11	11.15
Gpr18	2.60	H2B-1B	7.61	H2A-2a	27.18	Magee1	27.77	H3-1	25.93	Slc26a3	11.12
Hpca	2.51	H2A-1F	7.27	H2B-1a	26.90	H4	27.70	H3-1	25.29	Tubb1	10.89
Arid3c	2.50	Cmtm2	7.26	WASF1	26.85	H2B-1	27.54	Wdr93	24.56	PPHLN1	10.65
Jun	2.50	H2B-1	6.98	H2B-1	26.79	Egr2	27.32	H2B-1	24.07	Fhl4	10.51
Egr1	2.50	Csf2	6.75	H3-1	26.40	PRDM9	26.80	Egr2	23.58	Rnf43	10.37
ZNF540	2.45	H2A-1b	6.56	H2B-1	25.66	H3-1	26.34	Fos	23.31	LINE-1	10.20
Fam189a1	2.43	H2A-2a	6.30	H2A-1b	24.63	Hao1	25.79	Tnfaip3	20.93	H2A-1b	10.01
KLHDC1	2.40	Slc36a3	6.09	H4	22.39	Ucn2	24.97	H2B-1	20.71	Dbp	9.48
Snph	2.40	Ccn1	5.72	PRDM9	21.30	ZKSCAN4	24.73	Dusp8	20.43	H2A-1b	9.40
Hoxa2	2.36	H3-3	5.71	Egr2	21.18	H3-1	24.70	H2B-1	20.25	H4	8.93
Mzf1	2.31	H4	5.67	H2B-1	19.37	Fam131b	24.43	Znf429	20.06	HERVK_113	8.92
Znf764	2.29	H2A-2a	5.44	H3-1	18.64	VAPA	23.81	VAPA	19.93	St18	8.30
Slc15a3	2.24	H2B-1	5.42	H2B-1	18.46	Gpr83	23.13	Ikzf3	19.39	IIGP1	8.25
Wt1	2.18	H2B-1b	5.34	H3-1	17.64	Dusp8	21.60	Odad4	19.25	ERVK-7	8.14
Ppfia3	2.17	Slc10a1	5.22	Foxred2	17.52	Znf429	21.50	Atf3	17.52	Nr4a3	7.75
Zscan2	2.14	H2B-1	5.10	Xkr8	15.99	OR3A1	21.45	Filip1l	17.09	Vtcn1	7.75
H3-3	2.12	Dusp1	5.09	H3	15.78	H2B-1	20.87	Txnip	16.80	Znf728	7.56
LOC100758638	2.12	H2B-1a	4.87	Ccn1	15.54	Tmem71	20.86	H2A-1b	16.48	Pax3	7.39
Klf6	2.10	Akap5	4.77	Fam83e	15.00	Znf420	20.71	Cnnm1	16.47	Cyp2b1	7.36
Dusp1	2.07	H1-4	4.56	VAPA	14.97	H2A-1b	20.45	Dipk2a	16.20	H3-2	7.26
Fbxl22	2.03	Gpr156	4.54	H4	14.87	H2B-1	20.24	Egr1	15.02	Ush2a	7.23
Znf26	2.03	Rhob	4.24	Ano7	14.72	Ano7	19.85	Adamts1	14.56	Dgkg	7.17
SETMAR	2.01	Artn	3.90	Col9a3	14.65	Shisa2	19.54	Ptgs2	13.95	Pgpep1l	6.93

Supplementary table 4: 50 genes showing the highest upregulation in response to deoxynivalenol exposure of V79 cells at the 7 different timepoints (from T15 to T24). The fold change (in rows marked: fc) in gene expression relative to untreated cells (T0) is shown and illustrated using the Microsoft Excel conditional formatting tool. Immediate early genes are shown in green; histone genes are shown in blue. Note that fold gene changes are most pronounced in timepoints T60 (60 minutes) to T120 (120 minutes).

gene	fc T15/T0	gene	fc T30/T0	gene	fc T60/T0	gene	fc T90/T0	gene	fc T120/T0	gene	fc T24/T0
Akr7a1	5.72	RPL31	4.02	LOC118239522	6.33	Furin	6.97	Pigz	7.23	Spp1	44.06
Pllp	5.23	Tmem249	3.68	Lhfpl2	5.64	Atp6v1g2	4.80	Mroh6	6.72	Aqp1	21.63
Atp6v1g2	4.51	Akr7a1	3.62	Tmem249	4.29	LOC118239886	4.47	Inhbe	6.08	LOC100773795	21.26
Sptbn5	3.81	Atp6v1g2	3.42	Hoxb5	4.11	Ptpre	4.11	Furin	5.77	Rbm44	20.80
Tmem249	3.70	LOC118240007	3.23	ATXN7L3	4.07	Slc22a17	4.08	LOC107978969	5.57	Pcyt1b	16.15
Angptl6	3.42	L3mbtl1	3.18	Slc22a17	3.90	Tmem249	3.84	Kcns3	5.40	Ldlr	15.56
LOC118239522	3.41	Pcnx2	3.12	pou5f3	3.89	Mroh6	3.77	Nxpe1	5.23	Hsd11b1	15.06
Insl3	3.33	Csr1	3.11	Tmem117	3.72	Hoxb5	3.54	Pianp	4.77	Ano9	14.12
FGFR1OP2	3.25	Spaca6	2.93	Atp6v1g2	3.72	Prtg	3.42	Ddit4	4.67	Nxpe1	12.98
RALGAPA1	3.13	Shd	2.84	Igsf6	3.36	Lhfpl2	3.41	Slc22a17	4.32	F2rl2	12.79
Tnfrsf26	3.03	Eya1	2.81	Mmrn2	3.12	Ccdc159	3.20	Kcnj4	4.27	Zc4h2	12.78
Lhb	2.95	Nxnl1	2.78	LOC113836679	3.10	Csr1	3.20	LOC118239888	4.24	Egr3	12.28
Retn	2.90	LOC118239522	2.69	PTK7	3.01	Bnipl	3.00	Hspg2	3.98	Chst15	11.88
LRP1	2.86	Izumo4	2.68	Csr1	2.90	Prrt4	2.91	LOC100764240	3.96	Chl1	11.44
Sema4f	2.82	Cyp2j3	2.64	Rac3	2.87	Proc	2.87	Hoxb5	3.95	Dhcr24	11.40
Shd	2.78	PEX3	2.54	Pianp	2.77	Ctrl	2.86	Card9	3.93	Il10	11.10
Dnd1	2.69	Slc16a4	2.53	Mroh6	2.75	Adss1	2.86	Atoh8	3.68	Ddit4	10.79
Kcnk15	2.62	QCR6	2.53	LOC118239888	2.69	LOC100764240	2.83	Auts2	3.65	Slc7a11	10.62
Baiap3	2.56	Igsf6	2.52	Or8k22	2.53	LOC118239888	2.80	Megf8	3.60	Aass	10.59
Gpd1	2.53	Slc22a17	2.32	Rnf125	2.51	LOC107977858	2.76	Shc4	3.57	Hao2	10.46
Igfbp6	2.49	Kif5a	2.31	CUNH16orf90	2.45	Kcnn1	2.73	LOC103158941	3.45	Sh3tc1	9.96
Spaca6	2.46	INSC	2.30	CUNH11orf65	2.37	Prx	2.71	Fam78a	3.44	Lpin1	9.67
PAPOLA	2.44	ERVK-7	2.25	Izumo4	2.35	Hspg2	2.62	Sema6b	3.43	LOC103160853	9.62
Vil1	2.40	Pianp	2.25	Ddit4	2.34	Cdk3	2.60	C3H10orf82	3.42	LOC118237610	9.41
TGTP1	2.32	LRP1	2.23	Ccdc159	2.34	Zc3h12d	2.57	Lama5	3.40	Eya1	9.37
LOC103158541	2.30	Wdr97	2.22	Ankmy1	2.27	Hic1	2.52	Atp6v1g2	3.38	LOC100768856	9.25
LOC118239888	2.29	GSTT1	2.22	LOC100769895	2.27	Kcns3	2.50	Prtg	3.36	LOC100757534	9.16
Or8k22	2.24	LOC100764284	2.20	LOC114079438	2.26	Setd1b	2.50	Ankrd34a	3.33	Snx32	9.06
Slc39a5	2.23	MATCAP2	2.18	GRAMD1B	2.23	LOC100762393	2.49	Fut11	3.21	Serpini1	9.05
Ccdc159	2.17	LOC118239888	2.14	Trmu	2.20	Fut11	2.48	LOC100769912	3.21	Pnma1	8.88
Tsnaxip1	2.12	Sgca	2.10	Prtg	2.17	Il25	2.47	LOC100752894	3.21	Rhpn1	8.77
Slc22a17	2.12	Gng5	2.09	Slc35g6	2.16	Idh2	2.45	Ccdc3	3.06	Rtn2	8.71
Pigz	2.08	Rhof	2.08	GSTT1	2.15	CUNH8orf58	2.45	CUNH8orf58	3.05	Plb1	8.70
Rhbdl1	2.08	Fam219a	2.06	Dnah17	2.14	LOC100752166	2.45	Tfap4	3.03	Kyat3	8.47
Izumo4	2.07	Pla2g4e	2.04	Abtb1	2.09	Lama5	2.39	Trib3	3.01	LOC100754646	8.33
LOC118240029	2.02	LMWPTP	2.04	Vasn	2.08	LOC118239522	2.38	Rac3	2.98	LOC100750890	8.22
LOC118240007	2.01	Hsd11b1	2.00	Letm2	2.05	Rac3	2.37	Atoh8	2.97	Nrn1	8.12
				Syngap1	2.04	LOC100768894	2.37	Rnf145	2.88	CUNH11orf86	8.04
				Trim46	2.04	Megf8	2.36	Syngr3	2.88	Srpx2	7.99
				Fut11	2.03	Ankrd37	2.32	Tmem121	2.86	Tspan1	7.83
				Setd1b	2.01	Ager	2.31	LOC118239200	2.85	Pls1	7.81
						Zbtb42	2.28	Zc3h12d	2.85	Col18a1	7.78
						Cideb	2.28	Marchf9	2.85	Itih5	7.78
						Vasn	2.27	Hs3st6	2.80	Adgrf1	7.74
						LOC113836679	2.25	Ccdc181	2.78	Ssbp4	7.68
						LOC100753123	2.22	Bnipl	2.77	Mmp10	7.63
						Dicer1	2.22	Slc25a13	2.76	Nqo1	7.62
						Abtb1	2.19	Proc	2.76	Spaca6	7.56
						Sdcbp2	2.19	Sesn2	2.76	Sema3b	7.52
						Pla2g4e	2.18	LOC100762019	2.75	LOC100770323	7.50

Supplementary table 5: 50 genes showing the strongest downregulation in expression in response to deoxynivalenol exposure at the 7 different timepoints (from T15 to T24). The fold change in gene expression is shown and illustrated using the Microsoft Excel conditional formatting. Most substantially changed genes are shown in green. Note that fold gene changes are most pronounced at timepoint T24 (24 hours).

gene	av T15 FPKM	fc T15/T0	gene	T30 FPKM	fc T30/T0	gene	T60 FPKM	fc T60/T0	gene	T90 FPKM	fc T90/T0	gene	T120 FPKM	fc T120/T0	gene	T24 FPKM	fc T24/T0
Jun	36.26	2.50	Jun	172.42	11.90	Jun	578.22	39.91	Thbs1	1302.74	2.78	Thbs1	1815.86	3.88	Ubb	2292.87	2.08
Egr1	28.57	2.50	Egr1	171.52	15.00	H1-0	571.93	2.05	H3-3	954.20	2.40	H3-3	1046.28	2.63	SPRR1A	1032.91	3.33
Dusp1	23.44	2.07	Ccn1	101.64	5.72	Egr1	528.90	46.26	H1-0	722.28	2.59	H1-0	863.03	3.10	Plin2	726.03	4.65
Klf6	11.14	2.10	Klf6	60.24	2.41	Ier3	460.04	2.22	Jun	688.63	47.53	SPRR1A	760.83	2.45	H1-0	558.42	2.00
H1-2	8.31	8.46	Sgk1	58.90	2.04	Ubc	358.01	2.12	Tsc22d1	500.64	2.87	Tsc22d1	648.13	3.71	Ubc	399.01	2.36
PRC1	7.83	8.18	Txnip	58.33	2.51	Mt2	319.77	3.63	Egr1	492.45	43.07	Mt2	555.24	6.31	Tnfrsf12a	306.53	2.11
Znf26	3.53	2.03	Dusp1	57.53	5.09	Ccn1	276.16	15.54	Ier3	467.07	2.25	Ubc	496.71	2.94	Ifrd1	290.60	3.32
H3	2.64	7.55	H2B-1	55.74	5.42	H2B-1	275.74	26.79	Mt2	403.47	4.59	c-myc	463.96	3.98	Dnttip2	286.13	2.05
Fos	1.80	5.21	PHLDA1	54.02	2.09	c-myc	267.61	2.29	Ubc	396.14	2.35	Fosl1	460.70	4.26	Bloc1s2	252.07	2.44
H2A-1b	1.03	2.93	H2B-1	43.41	3.60	Mt1	261.02	3.15	c-myc	374.48	3.21	Areg	415.65	3.10	Ngdn	227.68	2.03
H3-3	0.81	2.12	H1-2	34.22	34.84	Fosl1	242.13	2.24	Txnip	362.54	15.58	Mt1	403.85	4.87	Nob1	220.22	2.81
INPP5F	0.73	2.12	Rhob	29.58	4.24	Txnip	235.07	10.10	Fosl1	324.41	3.00	Txnip	390.90	16.80	Cenpv	214.62	2.14
H3-1	0.73	6.74	Tnfaip3	18.45	2.06	Ifrd1	196.57	2.25	H2B-1	321.47	31.24	Fst	387.96	3.29	Gpx8	204.31	2.14
H3-1	0.65	6.55	Klf6	17.73	3.34	Errfi1	159.91	2.20	Areg	319.35	2.38	Tnfrsf12a	384.11	2.64	Wdr43	204.12	2.36
Gng13	0.61	3.27	Adamts1	15.39	2.75	Dusp1	145.69	12.89	Mt1	314.39	3.79	Jun	382.31	26.39	Gnl2	192.08	2.02
H4	0.61	3.74	Fos	13.53	39.18	PHLDA1	123.58	4.77	Tnfrsf12a	294.21	2.03	Ifrd1	335.39	3.83	Ankrd1	178.42	4.73
SETMAR	0.53	2.01	Csrnp1	11.93	2.28	Tiparp	116.50	2.14	Ifrd1	263.40	3.01	H2B-1	314.82	30.59	Snw1	160.17	2.06
Znf764	0.46	2.29	Tmsb4x	10.49	2.36	Myc	106.15	2.28	Ccn1	246.56	13.88	Mcl1	302.73	2.10	Ppp1r12a	158.57	2.65
H3-1	0.40	4.96	Smad7	8.77	2.01	Klf6	103.42	4.13	Btg1	230.76	2.45	Btg1	302.46	3.21	Prpf38a	153.64	2.01
H2A-1b	0.37	3.16	Ptgs2	8.49	2.35	Junb	98.36	3.74	Errfi1	198.95	2.74	Ankrd1	291.63	7.73	Cdc42ep3	152.07	2.81
ZNF146	0.36	2.72	H2B-2f	6.07	3.32	Ankrd1	98.21	2.60	PHLDA1	188.31	7.28	Errfi1	270.31	3.72	Ccdc137	145.77	2.47
Arid3c	0.35	2.50	Gadd45g	5.90	2.13	Rnd3	92.45	3.03	Ankrd1	179.20	4.75	PHLDA1	259.25	10.02	Lamb1	142.47	2.47
Mzf1	0.34	2.31	H2B-1b	5.78	5.34	Dusp5	86.45	2.86	Dusp1	161.75	14.31	Cdc42ep3	222.17	4.10	Gpatch4	142.27	2.83
Hpca	0.29	2.51	L-110308656	4.81	13.72	H1-2	85.83	87.37	Tiparp	158.66	2.92	Rnd3	218.00	7.14	Cited2	140.72	2.30
Zscan2	0.22	2.14	Znf26	4.49	2.58	Ppp1r15a	85.78	3.31	Tnfaip3	157.55	17.59	Tnfaip3	187.45	20.93	RASSF1	139.47	2.66
KLHDC1	0.22	2.40	H3	3.70	10.56	Insig1	83.10	2.75	Myc	151.92	3.27	Myc	185.73	3.99	Tmx2	138.35	2.13
Slc15a3	0.21	2.24	Atf3	3.48	2.66	Has2	78.68	2.69	Rnd3	150.81	4.94	Egr1	171.80	15.02	Rnaset2	135.97	2.01
MAPT	0.20	2.85	H2B-1a	3.41	4.87	Tnfaip3	77.05	8.60	Klf6	146.12	5.84	Has2	163.98	5.60	Rps27a	135.26	2.08
Wt1	0.20	2.18	H2B-1	3.25	5.10	Topors	76.92	2.48	Cdc42ep3	138.05	2.55	Tiparp	160.03	2.94	Cdt1	134.67	3.15
Ypel2	0.19	3.72	Dusp8	3.16	3.15	H2B-1	73.70	6.10	Junb	133.19	5.07	Cited2	151.06	2.47	Exosc2	131.25	2.23
H2B-1	0.19	4.73	Hrh1	2.92	2.12	Sgk1	70.79	2.45	H1-2	130.35	132.70	Csf1	148.16	6.00	Mrps18b	125.69	2.12
Snph	0.18	2.40	H2A-2a	2.83	6.30	Chac1	68.55	2.36	Ppp1r15a	115.14	4.45	Ets1	146.47	2.39	Testin-2	125.27	2.19
H2A-1b	0.18	9.71	Pax3	2.77	2.02	Rcan1	56.48	2.15	Has2	111.52	3.81	Klf6	142.36	5.69	Ccdc137	120.08	2.54
Ppfia3	0.17	2.17	H3-1	2.49	23.01	Fos	54.98	159.20	Id3	111.33	2.14	Ppp1r15a	140.69	5.43	Fcf1	118.55	2.03
Dnai2	0.14	3.88	Klf2	2.45	2.30	Znf655	54.56	2.25	Dusp5	93.85	3.11	Junb	140.19	5.33	Hcfc1r1	117.71	2.56
Gpr18	0.14	2.60	H3-3	2.17	5.71	Sertad1	50.45	2.65	Chac1	90.48	3.11	H1-2	134.91	137.34	Tmem11	117.02	2.52
ZNF540	0.12	2.45	H2A-1b	1.96	30.54	Klf10	45.42	3.55	DSG2	90.04	9.40	Ccn1	132.63	7.47	PPHLN1	117.02	2.21
Mcidas	0.11	8.53	H2B-1	1.84	6.98	Nfkbia	45.07	2.56	Nfkbia	88.24	5.02	Rcan1	127.93	4.87	Wdr74	116.63	2.46
H4	0.10	9.21	Egr2	1.72	3.55	Ngf	44.93	2.04	Sgk1	86.16	2.98	Srxn1	117.43	2.55	Sqstm1	116.19	2.08
Fbxl22	0.08	2.03	Znf54	1.64	2.24	Csrnp1	43.17	8.24	Topors	85.04	2.74	DSG2	113.90	11.89	Prrx1	115.67	2.34
Gpr1	0.08	12.58	Fosb	1.64	3.59	Plk2	43.02	3.66	Rcan1	84.61	3.22	Thbd	113.18	3.34	Txnip	114.45	4.92
Kcnj12	0.08	3.54	H2B-1M	1.58	7.62	Has2	42.89	2.74	Csf1	74.86	3.03	Sgk1	111.64	3.87	Rnd3	114.38	3.74
Fam189a1	0.08	2.43	H1-4	1.48	4.56	Adamts1	40.70	7.26	Insig1	71.00	2.35	Lif	103.14	10.24	Srp68	111.04	2.30
Nat8f1	0.07	3.52	H2A-2a	1.29	5.44	Ppp1r10	40.55	4.31	Idi1	70.01	2.05	Klf10	99.58	7.77	Ggnbp2	106.68	2.08
FHL1	0.06	3.98	H3-1	1.28	12.97	Cxcl1	39.86	8.34	Amotl2	69.82	2.65	Has2	98.09	6.26	Trmt10c	105.89	2.14
Hoxa2	0.06	2.36	Znf709	1.14	2.08	SLFN14	39.74	2.13	Znf655	68.37	2.82	Gadd45b	94.79	4.15	Xbp1	105.10	2.55
Calcr	0.05	3.70	H2B-1B	1.13	28.69	DSG2	38.78	4.05	Sertad1	68.20	3.58	Plk2	93.67	7.97	Riok2	102.26	2.30
Marveld2	0.03	3.33	H2B-1	1.11	7.78	Hes1	36.41	3.04	Plk2	68.18	5.80	Ormdl1	90.35	4.62	Dctn4	100.96	3.08
Zscan18	0.02	8.73	ACOT5-like	0.95	2.71	Rhob	36.16	5.18	Cxcl1	68.06	14.25	Dusp5	87.89	2.91	Mrps30	100.21	2.23
Dnah9	0.01	11.94	Gab3	0.93	2.30	Rpp38	33.74	2.13	H2B-1	67.97	5.63	Dusp1	87.32	7.72	Zfr	99.89	2.47

Supplementary table 6: 50 genes showing the highest expression (in FPKM) of significantly ($p<0.05$) and substantially (fold change >2) upregulated genes in V79 cells in response to deoxynivalenol exposure at the 7 different timepoints (from T15 to T24). The FPKM value for the different genes is shown (av. FPKM) as is the fold change in gene expression (fc). Both columns are illustrated using the Microsoft Excel conditional formatting tool. Immediate early genes are shown in green; histone genes are shown in blue.

gene	fc T15/T0	T15 FPKM	gene	fc T30/T0	T30 FPKM	gene	fc T60/T0	T60 FPKM	gene	fc T90/T0	T90 FPKM	gene	fc T120/T0	T120 FPKM	gene	fc T24/T0	T24 FPKM
Akr7a1	5.72	0.03	RPL31	4.02	0.02	LOC118239522	6.33	0.06	Furin	6.97	0.05	Pigz	7.23	0.02	Spp1	44.06	0.04
Pllp	5.23	0.04	Tmem249	3.68	0.10	Lhfpl2	5.64	0.01	Atp6v1g2	4.80	0.38	Mroh6	6.72	0.03	Aqp1	21.63	0.04
Atp6v1g2	4.51	0.41	Akr7a1	3.62	0.04	Tmem249	4.29	0.09	LOC118239886	4.47	0.16	Inhbe	6.08	0.03	LOC100773795	21.26	1.21
Sptbn5	3.81	0.01	Atp6v1g2	3.42	0.54	Hoxb5	4.11	0.09	Ptpre	4.11	0.03	Furin	5.77	0.06	Rbm44	20.80	0.03
Tmem249	3.70	0.10	LOC118240007	3.23	0.39	ATXN7L3	4.07	1.29	Slc22a17	4.08	0.35	LOC107978969	5.57	0.04	Pcyt1b	16.15	0.01
Angptl6	3.42	0.03	L3mbtl1	3.18	0.02	Slc22a17	3.90	0.36	Tmem249	3.84	0.10	Kcns3	5.40	1.23	Ldlr	15.56	1.66
LOC118239522	3.41	0.11	Pcnx2	3.12	0.03	pou5f3	3.89	0.19	Mroh6	3.77	0.05	Nxpe1	5.23	0.04	Hsd11b1	15.06	0.02
Insl3	3.33	0.06	Csr1	3.11	0.11	Tmem117	3.72	0.05	Hoxb5	3.54	0.11	Pianp	4.77	0.05	Ano9	14.12	0.03
FGFR1OP2	3.25	0.12	Spaca6	2.93	0.17	Atp6v1g2	3.72	0.49	Prtg	3.42	0.47	Ddit4	4.67	1.41	Nxpe1	12.98	0.02
RALGAPA1	3.13	0.21	Shd	2.84	0.08	Igsf6	3.36	0.05	Lhfpl2	3.41	0.02	Slc22a17	4.32	0.33	F2rl2	12.79	0.02
Tnfrsf26	3.03	0.05	Eya1	2.81	0.10	Mmrn2	3.12	0.02	Ccdc159	3.20	0.22	Kcnj4	4.27	0.58	Zc4h2	12.78	0.15
Lhb	2.95	0.16	Nxnl1	2.78	0.02	LOC113836679	3.10	0.30	Csr1	3.20	0.11	LOC118239888	4.24	0.19	Egr3	12.28	0.07
Retn	2.90	0.14	LOC118239522	2.69	0.14	PTK7	3.01	0.10	Bnipl	3.00	0.06	Hspg2	3.98	0.79	Chst15	11.88	0.02
LRP1	2.86	0.03	Izumo4	2.68	0.77	Csr1	2.90	0.12	Prrt4	2.91	0.07	LOC100764240	3.96	1.37	Chl1	11.44	0.06
Sema4f	2.82	0.03	Cyp2j3	2.64	0.04	Rac3	2.87	0.43	Proc	2.87	0.07	Hoxb5	3.95	0.10	Dhcr24	11.40	4.65
Shd	2.78	0.08	PEX3	2.54	0.09	Pianp	2.77	0.09	Ctrl	2.86	0.12	Card9	3.93	0.05	Il10	11.10	0.10
Dnd1	2.69	0.34	Slc16a4	2.53	0.34	Mroh6	2.75	0.07	Adss1	2.86	0.05	Atoh8	3.68	0.50	Ddit4	10.79	0.61
Kcnk15	2.62	0.10	QCR6	2.53	0.18	LOC118239888	2.69	0.30	LOC100764240	2.83	1.92	Auts2	3.65	0.03	Slc7a11	10.62	0.14
Baiap3	2.56	0.04	Igsf6	2.52	0.07	Or8k22	2.53	0.08	LOC118239888	2.80	0.29	Megf8	3.60	1.78	Aass	10.59	0.38
Gpd1	2.53	0.02	Slc22a17	2.32	0.61	Rnf125	2.51	0.03	LOC107977858	2.76	0.23	Shc4	3.57	0.01	Hao2	10.46	0.01
Igfbp6	2.49	0.02	Kif5a	2.31	0.04	CUNH16orf90	2.45	0.06	Kcnn1	2.73	0.03	LOC103158941	3.45	0.24	Sh3tc1	9.96	0.06
Spaca6	2.46	0.20	INSC	2.30	0.22	CUNH11orf65	2.37	0.09	Prx	2.71	0.01	Fam78a	3.44	0.15	Lpin1	9.67	0.01
PAPOLA	2.44	0.04	ERVK-7	2.25	0.40	Izumo4	2.35	0.88	Hspg2	2.62	1.19	Sema6b	3.43	0.73	LOC103160853	9.62	0.01
Vil1	2.40	0.07	Pianp	2.25	0.11	Ddit4	2.34	2.81	Cdk3	2.60	0.03	C3H10orf82	3.42	0.03	LOC118237610	9.41	0.04
TGTP1	2.32	0.02	LRP1	2.23	0.04	Ccdc159	2.34	0.31	Zc3h12d	2.57	0.39	Lama5	3.40	5.52	Eya1	9.37	0.03
LOC103158541	2.30	3.03	Wdr97	2.22	0.08	Ankmy1	2.27	0.16	Hic1	2.52	1.99	Atp6v1g2	3.38	0.54	LOC100768856	9.25	0.08
LOC118239888	2.29	0.35	GSTT1	2.22	2.44	LOC100769895	2.27	0.39	Kcns3	2.50	2.66	Prtg	3.36	0.48	LOC100757534	9.16	1.62
Or8k22	2.24	0.09	LOC100764284	2.20	0.17	LOC114079438	2.26	0.88	Setd1b	2.50	0.64	Ankrd34a	3.33	0.34	Snx32	9.06	0.04
Slc39a5	2.23	0.47	MATCAP2	2.18	0.07	GRAMD1B	2.23	0.12	LOC100762393	2.49	0.04	Fut11	3.21	1.23	Serpini1	9.05	0.03
Ccdc159	2.17	0.33	LOC118239888	2.14	0.38	Trmu	2.20	0.79	Fut11	2.48	1.60	LOC100769912	3.21	0.83	Pnma1	8.88	0.03
Tsnaxip1	2.12	0.02	Sgca	2.10	0.08	Prtg	2.17	0.73	Il25	2.47	0.06	LOC100752894	3.21	0.12	Rhpn1	8.77	0.04
Slc22a17	2.12	0.67	Gng5	2.09	0.53	Slc35g6	2.16	0.18	Idh2	2.45	1.53	Ccdc3	3.06	0.18	Rtn2	8.71	0.29
Pigz	2.08	0.07	Rhof	2.08	0.29	GSTT1	2.15	2.51	CUNH8orf58	2.45	0.86	CUNH8orf58	3.05	0.69	Plb1	8.70	0.06
Rhbdl1	2.08	0.18	Fam219a	2.06	0.33	Dnah17	2.14	0.01	LOC100752166	2.45	0.09	Tfap4	3.03	4.70	Kyat3	8.47	1.79
Izumo4	2.07	1.00	Pla2g4e	2.04	0.11	Abtb1	2.09	0.73	Lama5	2.39	7.84	Trib3	3.01	3.13	LOC100754646	8.33	1.18
LOC118240029	2.02	0.23	LMWPTP	2.04	0.55	Vasn	2.08	0.25	LOC118239522	2.38	0.16	Rac3	2.98	0.41	LOC100750890	8.22	4.54
LOC118240007	2.01	0.63	Hsd11b1	2.00	0.17	Letm2	2.05	0.24	Rac3	2.37	0.52	Atoh8	2.97	3.13	Nrn1	8.12	1.95
						Syngap1	2.04	0.38	LOC100768894	2.37	0.09	Rnf145	2.88	15.27	CUNH11orf86	8.04	0.30
						Trim46	2.04	0.80	Megf8	2.36	2.71	Syngr3	2.88	0.17	Srpx2	7.99	0.13
						Fut11	2.03	1.94	Ankrd37	2.32	1.51	Tmem121	2.86	0.80	Tspan1	7.83	0.08
						Setd1b	2.01	0.80	Ager	2.31	0.77	LOC118239200	2.85	0.09	Pls1	7.81	0.15
									Zbtb42	2.28	1.61	Zc3h12d	2.85	0.35	Col18a1	7.78	0.23
									Cideb	2.28	0.12	Marchf9	2.85	3.01	Itih5	7.78	0.01
									Vasn	2.27	0.23	Hs3st6	2.80	0.31	Adgrf1	7.74	0.03
									LOC113836679	2.25	0.41	Ccdc181	2.78	0.11	Ssbp4	7.68	0.87
									LOC100753123	2.22	0.88	Bnipl	2.77	0.06	Mmp10	7.63	0.39
									Dicer1	2.22	4.77	Slc25a13	2.76	4.40	Nqo1	7.62	2.00
									Abtb1	2.19	0.69	Proc	2.76	0.07	Spaca6	7.56	0.06
									Sdcbp2	2.19	0.42	Sesn2	2.76	1.60	Sema3b	7.52	0.17
									Pla2g4e	2.18	0.10	LOC100762019	2.75	0.18	LOC100770323	7.50	1.25

Supplementary table 7: 50 genes showing the highest expression (in FPKM) of significantly (p<0.05) and substantially (fold change >2) downregulated genes in response to deoxynivalenol exposure at the 7 different timepoints (from T15 to T24). The FPKM value for the different gene is shows as is the fold change in gene expression. The FPKM value for the different genes is shown (av. FPKM) as is the fold change in gene expression (fc). Both columns are illustrated using the Microsoft Excel conditional formatting tool. Note that expression of the most downregulated genes is typically low.

timepoint	source	term_name	term ID	[-]log10 p-value
T30	GO:MF	DNA-binding transcription factor activity, RNA polymerase II-specific	GO:0000981	4.08
	GO:MF	DNA binding	GO:0003677	3.40
	GO:MF	R-SMAD binding	GO:0070412	1.81
	GO:MF	glial cell-derived neurotrophic factor receptor binding	GO:0030116	1.70
	GO:BP	positive regulation of transcription by RNA polymerase II	GO:0045944	2.99
	GO:BP	muscle organ development	GO:0007517	2.87
	GO:BP	response to oxidative stress	GO:0006979	2.82
	GO:BP	positive regulation of biological process	GO:0048518	2.57
	GO:BP	tissue development	GO:0009888	2.51
	GO:BP	cellular response to chemical stimulus	GO:0070887	2.44
	GO:BP	skeletal muscle cell differentiation	GO:0035914	2.43
	GO:BP	regulation of cellular biosynthetic process	GO:0031326	1.50
	GO:CC	transcription factor AP-1 complex	GO:0035976	1.78
	GO:CC	protein-DNA complex	GO:0032993	1.78
T60	GO:MF	DNA-binding transcription factor activity	GO:0003700	8.78
	GO:MF	sequence-specific DNA binding	GO:0043565	7.91
	GO:MF	binding	GO:0005488	4.81
	GO:MF	MAP kinase tyrosine/serine/threonine phosphatase activity	GO:0017017	4.00
	GO:MF	SMAD binding	GO:0046332	1.45
	GO:BP	regulation of transcription by RNA polymerase II	GO:0006357	7.80
	GO:BP	intracellular signal transduction	GO:0035556	2.67
	GO:BP	regulation of cell adhesion	GO:0030155	2.53
	GO:BP	programmed cell death	GO:0012501	1.95
	GO:BP	response to lipid	GO:0033993	1.94
	GO:BP	cell surface receptor signaling pathway	GO:0007166	1.71
	GO:BP	reproductive process	GO:0022414	1.47
	GO:CC	transcription regulator complex	GO:0005667	4.26
T90	GO:MF	DNA binding	GO:0003677	13.05
	GO:MF	structural constituent of chromatin	GO:0030527	8.47
	GO:MF	DNA-binding transcription factor activity	GO:0003700	6.28
	GO:MF	binding	GO:0005488	6.23
	GO:MF	MAP kinase tyrosine/serine/threonine phosphatase activity	GO:0017017	4.45
	GO:MF	protein heterodimerization activity	GO:0046982	2.16
	GO:MF	adenylate cyclase binding	GO:0008179	1.63
	GO:MF	metal ion binding	GO:0046872	1.60
	GO:BP	tissue development	GO:0009888	12.11
	GO:BP	positive regulation of programmed cell death	GO:0043068	4.45
	GO:BP	p38MAPK cascade	GO:0038066	2.66
	GO:BP	smooth muscle cell proliferation	GO:0048659	2.48
	GO:BP	fat pad development	GO:0060613	2.23
	GO:BP	inflammatory response	GO:0006954	1.91
	GO:BP	regulation of adaptive immune response	GO:0002819	1.90
	GO:BP	reproductive process	GO:0022414	1.64
	GO:BP	regulation of T-helper cell differentiation	GO:0045622	1.50
	GO:BP	immune system process	GO:0002376	1.37
	GO:BP	negative regulation of ERK1 and ERK2 cascade	GO:0070373	1.34
	GO:CC	nucleosome	GO:0000786	9.81
	GO:CC	transcription factor AP-1 complex	GO:0035976	2.03

**T120**	GO:MF	DNA binding	GO:0003677	13.88
	GO:MF	DNA-binding transcription factor activity	GO:0003700	13.05
	GO:MF	binding	GO:0005488	11.01
	GO:MF	MAP kinase tyrosine/serine/threonine phosphatase activity	GO:0017017	3.81
	GO:MF	metal ion binding	GO:0046872	1.90
	GO:MF	glycogen binding	GO:2001069	1.30
	GO:BP	regulation of primary metabolic process	GO:0080090	24.94
	GO:BP	adaptive immune response based on somatic recombination of immune receptors built from immunoglobulin superfamily domains	GO:0002460	2.79
	GO:BP	growth	GO:0040007	1.78
	GO:BP	reproductive process	GO:0022414	1.71
	GO:BP	locomotion	GO:0040011	1.56
	GO:BP	platelet-derived growth factor receptor signaling pathway	GO:0048008	1.45
	GO:BP	transforming growth factor beta receptor superfamily signaling pathway	GO:0141091	1.43
	GO:CC	nucleoplasm	GO:0005654	5.09
	GO:CC	transcription regulator complex	GO:0005667	3.27
	GO:CC	protein-DNA complex	GO:0032993	3.07
	GO:CC	extracellular matrix	GO:0031012	2.40
	GO:CC	nucleus	GO:0005634	2.12
	GO:CC	piccolo histone acetyltransferase complex	GO:0032777	1.99
	GO:CC	death-inducing signalling complex	GO:0031264	1.60
	GO:CC	transcription factor AP-1 complex	GO:0035976	1.60
**T24**	GO:MF	DNA binding	GO:0003677	13.09
	GO:MF	transcription regulator activity	GO:0140110	12.49
	GO:MF	binding	GO:0005488	9.69
	GO:MF	zinc ion binding	GO:0008270	2.80
	GO:MF	structural constituent of chromatin	GO:0030527	1.72
	GO:MF	S-adenosylmethionine-dependent methyltransferase activity	GO:0008757	1.64
	GO:MF	ubiquitin-protein transferase activity	GO:0004842	1.33
	GO:BP	regulation of nucleobase-containing compound metabolic process	GO:0019219	20.24
	GO:BP	intracellular signal transduction	GO:0035556	1.89
	GO:BP	cellular response to chemical stimulus	GO:0070887	1.52
	GO:BP	response to organic substance	GO:0010033	1.48
	GO:CC	nucleoplasm	GO:0005654	18.23
	GO:CC	protein-DNA complex	GO:0032993	7.73

Supplementary table 8: Pathway analysis of genes upregulated significantly (p<0.05) and substantially (fold change >2) in response to treatment of V79 cells with DON at time points T30 to T24. No significant changes of pathways were detected at time point T15 (15 min. exposure to DON). Pathway analysis was carried out in G-Profiler (which recognizes Chinese Hamster gene locus designations). Pathways are grouped by "molecular function" (MF), "biological process" (BP) and "cellular compartment" (CC). The corresponding GO terms and the negative log10 of the p-value are shown for all pathways. The pathways shown are all the pathways which are classed as true by the G-Profiler software. DNA binding (GO-term 0003677) or binding (GO-term 0005488) are found at all timepoints (marked in blue). The GO-terms 0017017 (MAP kinase activity; marked in green) and 0035976 (transcription factor AP-1 complex; marked in yellow) are found significantly regulated during timepoints T60, T90 and T120 when the highest amplitudes of Fos/Jun cell stress pathway activation are found in V79 cells exposed to DON.

T15 up

Term	Adj. P-value	LINCS 1000 - CRISPR KO genes
PDXK Down	0.328	Pyridoxal Kinase
ABI3 Down	0.328	ABI Family Member 3
APOBEC3G Down	0.328	Apolipoprotein B MRNA Editing Enzyme Catalytic Subunit 3G
CDC42BPB Up	0.328	CDC42 Binding Protein Kinase Beta
SIPA1L1 Up	0.328	Signal Induced Proliferation Associated 1 Like 1
CLK4 Up	0.328	CDC Like Kinase 4
RPP40 Down	0.328	Ribonuclease P/MRP Subunit P40
PSG1 Up	0.328	Pregnancy Specific Beta-1-Glycoprotein 1
ZNF646 Up	0.328	Zinc Finger Protein 646
GNAI2 Down	0.328	G Protein Subunit Alpha I2

T15 down

Term	Adj. P-value	LINCS 1000 - CRISPR KO genes
BCL2L14 Up	0.372	
DDX58 Down	0.372	
BOP1 Down	0.372	
HLTF Down	0.372	
CCR1 Down	0.372	
SPNS2 Down	0.372	
STK25 Up	0.372	
ACO2 Up	0.372	
MAP4K1 ACTN4 Up	0.372	
NAT1 Down	0.372	

T30 up

Term	Adj. P-value	LINCS 1000 - CRISPR KO genes
ARSG Down	5.00E-05	Arylsulfatase G
MAP3K8 Down	5.00E-05	Mitogen-Activated Protein Kinase Kinase Kinase 8
CHEK1 Up	5.00E-05	Checkpoint Kinase 1
KCNT2 Up	5.00E-05	Potassium Sodium-Activated Channel Subfamily T Member 2
ABI3 Down	5.00E-05	ABI Family Member 3
PDCD10 Up	5.00E-05	Programmed Cell Death 10
PLCG2 Down	3.07E-04	Phospholipase C Gamma 2
SYN1 Down	3.07E-04	Synapsin I
MS4A4A Down	3.07E-04	Membrane Spanning 4-Domains A4A
CLP1 Down	3.07E-04	Cleavage Factor Polyribonucleotide Kinase Subunit 1

T30 down

Term	Adj. P-value	LINCS 1000 - CRISPR KO genes
PMEPA1 Up	0.372	
WARS2 Down	0.372	
SLC44A1 Up	0.372	
DGAT1 Down	0.372	
GCH1 Up	0.372	
SLC11A1 Up	0.372	
MUC20 Down	0.372	
NPRL2 Down	0.372	
CAMTA2 Up	0.372	
SKIV2L Up	0.372	

T60 up

Term	Adj. P-value	LINCS 1000 - CRISPR KO genes
CHEK1 Up	2.20E-12	Checkpoint Kinase 1
RFTN2 Down	7.81E-12	Raftlin Family Member 2
DGKH Up	4.37E-11	Diacylglycerol Kinase Eta
MAP4K1 Down	4.37E-11	Mitogen-Activated Protein Kinase Kinase 8
RRM1 Up	4.37E-11	Ribonucleotide Reductase Catalytic Subunit M1
C2 Down	5.92E-11	complement protein C2
ARSG Down	1.26E-10	Arylsulfatase G
MS4A4A Down	2.51E-10	Membrane Spanning 4-Domains A4A
LPIN2 Down	2.51E-10	Lipin 2
EMB Down	2.51E-10	Embigin

T60 down

Term	Adj. P-value	LINCS 1000 - CRISPR KO genes
IL1RL1 Down	0.403	
SLC11A1 Up	0.403	
DNMT3A Up	0.403	
SLC7A4 Down	0.403	
KIF19 Up	0.403	
SNX20 Down	0.403	
SARS Up	0.403	
SDC2 Down	0.403	
ATP5C1 Up	0.403	
MAP3K6 Down	0.403	

T90 up

Term	Adj. P-value	LINCS 1000 - CRISPR KO genes
ADAR Up	2.32E-11	Adenosine Deaminase RNA Specific
ARSG Down	6.16E-11	Arylsulfatase G
RFTN2 Down	2.83E-10	Raftlin Family Member 2
PIP4K2C Down	2.83E-10	Phosphatidylinositol-5-Phosphate 4-Kinase Type 2 Gamma
CHEK1 Up	1.56E-09	Checkpoint Kinase 1
RRM1 Up	1.79E-09	Ribonucleotide Reductase Catalytic Subunit M1
APOLD1 Down	1.80E-09	Apolipoprotein L Domain Containing 1
LPIN2 Down	1.80E-09	Lipin 2
EMB Down	1.80E-09	Embigin
MAP4K1 Down	5.13E-09	Mitogen-Activated Protein Kinase Kinase Kinase 8

T90 down

Term	Adj. P-value	LINCS 1000 - CRISPR KO genes
KCNK7 Down	0.581	
RUSC1 Down	0.581	
HLA-DOB Down	0.581	
CD209 Up	0.581	
FCGR1A Up	0.581	
SOCS5 Up	0.581	
ATF3 Down	0.581	
PPP1R15A Up	0.581	
NAT1 Down	0.581	
TNIP1 Down	0.581	

T120 up

Term	Adj. P-value	LINCS 1000 - CRISPR KO genes
ARSG Down	4.33E-13	Arylsulfatase G
ADAR Up	1.84E-12	Adenosine Deaminase RNA Specific
AP5B1 Down	8.15E-11	Adaptor Related Protein Complex 5 Subunit Beta 1
RPL19 Up	8.15E-11	Ribosomal Protein L19
DOCK4 Down	4.07E-10	Dedicator Of Cytokinesis 4
DAZL Down	1.27E-09	Deleted In Azoospermia Like
DSCAM Down	1.73E-09	DS Cell Adhesion Molecule
RFTN2 Down	2.64E-09	Raftlin Family Member 2
CHEK1 Up	2.64E-09	Checkpoint Kinase 1
ARPC2 Down	2.64E-09	Actin Related Protein 2/3 Complex Subunit 2

T120 down

Term	Adj. P-value	LINCS 1000 - CRISPR KO genes
IL1RL2 Down	0.603	
CLECL1 Down	0.603	
IQSEC3 Up	0.712	
CCL4 Up	0.712	
SETD2 Up	0.712	
HUNK Down	0.712	
DGKB Up	0.712	
CDK5R1 Down	0.712	
ETNK2 Up	0.712	
ITGA6 Down	0.712	

T24 up

Term	Adj. P-value	LINCS 1000 - CRISPR KO genes
RPL13 Up	0.74	Ribosomal Protein L13a
RPL19 Up	1.00	Ribosomal Protein L19
TTK Up	1.00	TTK Protein Kinase
RPS6KL1 Up	1.00	Ribosomal Protein S6 Kinase Like 1
RRP15 Up	1.00	Ribosomal RNA Processing 15 Homolog
VCP Up	1.00	Valosin Containing Protein/Transitional Endoplasmic Reticulum ATPase
NFS1 Up	1.00	NFS1 Cysteine Desulfurase
ARPC2 Down	1.00	Actin Related Protein 2/3 Complex Subunit 2
ME2 Down	1.00	Malic Enzyme 2
RPL37A Up	1.00	Ribosomal Protein L37a

T24 down

Term	Adj. P-value	LINCS 1000 - CRISPR KO genes
RPS17 Down	4.46E-10	Ribosomal Protein S17
RPS6 Down	4.27E-08	Ribosomal Protein S6
LDLR Up	9.64E-08	Low Density Lipoprotein Receptor
HMGCR Up	5.34E-06	3-Hydroxy-3-Methylglutaryl-CoA Reductase
EEF2 Down	1.07E-05	Eukaryotic Translation Elongation Factor 2
CD276 Down	1.15E-05	CD276
PRM1 Down	1.15E-05	Protamine 1
SERPING1 Up	3.31E-05	Serpin Family G Member 1/Plasma Protease C1 Inhibitor
EIF2B4 Up	6.51E-05	Eukaryotic Translation Initiation Factor 2B Subunit Delta
IFI30 Up	7.07E-05	IFI30 Lysosomal Thiol Reductase

Supplementary table 9: Similarities of gene expression changes in V79 cells treated with deoxynivalenol (DON) to other transcriptome datasets in the LINCS 1000 project. The genes changed significantly (P<0.05; fold change >2) in V79 cells in response to DON treatment for 15 min. (T15), 30 min. (T30), 60 min. (T60), 90 min. (T90), 120 min. (T120) or 24 hours (T24) were analysed in the EnrichR software suite (https://maayanlab.cloud/Enrichr/). DON responsive gene expression profiles were compared to available CRISPR knock-out cell transcriptomes. The top 10 transcriptome profiles identified for the different treatment periods are shown (genes upregulated in response to DON treatment are shown on left hand side, and genes downregulated in response to DON treatment are shown on right hand side for the 6 experimental timepoints) together with the p-values of the transcriptome similarities. Genes are ordered by p-values. CRISPR knock-out profiles for genes involved in cell proliferation, MAP kinase signalling and ribosomal function are marked in blue, red and green, respectively.

T15 up

Term	Adj. P-value	description of LINCS 1000 drug
Narciclasine Up	0.017	plant alkaloid, translation inhibitor
ST-4029573 Up	0.017	synthetic drug, unknown mechanism
SF-11006-A1 Down	0.017	synthetic drug, unknown mechanism
Tipiracil Down	0.017	synthetic thymidine phosphorylase inhibitor
GALR2 M617 Up	0.017	synthetic galanin receptor agonist
TL-HRAS-61 Up	0.017	chemical proble for ras analysis
Menadione Up	0.017	synthetic vitamin K analogue
CHEMBL-399379 Up	0.031	synthetic drug, unknown mechanism
Verrucarin-A Up	0.031	Myrothecium trichothecene mycotoxin
Cymarin Up	0.031	Apocynum glycoside, ATPase inhibitor

T30 up

Term	Adj. P-value	description of LINCS 1000 drug
Okadaic-Acid Up	2.00E-12	dinoflagellate neurotoxin, phosphatase inhibitor
NSC-663284 Up	2.22E-10	synthetic CDC25 phosphatase inhibitor
GALR2 M617 Up	2.22E-10	synthetic galanin receptor agonist
Helveticoside Up	2.56E-09	flixweed glycoside with unknown target
Narciclasine Up	1.98E-08	plant alkaloid, translation inhibitor
Periplocymarin Up	2.46E-08	silk vine glyoside, ATPase inhibitor
TL-HRAS-61 Up	2.46E-08	chemical proble for ras analysis
Anisomycin Up	1.78E-07	Streptomyces toxin, translation-elongation inhibitor
Cercosporin Up	1.78E-07	Cercosporin toxin, oxidative stressor
Cinobufagin Up	1.78E-07	toad toxin, induction of cell cycle arrest

T60 up

Term	Adj. P-value	description of LINCS 1000 drug
Narciclasine Up	1.40E-27	plant alkaloid, translation inhibitor
Okadaic-Acid Up	3.44E-27	dinoflagellate neurotoxin, phosphatase inhibitor
Anisomycin Up	3.12E-23	Streptomyces toxin, translation-elongation inhibitor
NSC-663284 Up	3.75E-23	synthetic CDC25 phosphatase inhibitor
Cercosporin Up	2.35E-22	Cercosporin toxin, oxidative stressor
I-606051 Up	2.35E-22	synthetic anti-proliferative drug, unknown mechanism
Ryuvidine Up	6.97E-20	synthetic inhibitor of histone methylation
I-606061 Up	2.30E-19	synthetic anti-proliferative drug, unknown mechanism
Amanitin Down	6.08E-19	inhibitor of RNA PolII, but not RNA PolI; i.e. ribosomal RNA is transcribed
STK-397047 Up	6.25E-19	synthetic drug, unknown mechanism

T90 up

Term	Adj. P-value	description of LINCS 1000 drug
Narciclasine Up	7.65E-29	plant alkaloid, translation inhibitor
Anisomycin Up	9.92E-27	Streptomyces toxin, translation-elongation inhibitor
Okadaic-Acid Up	2.47E-21	dinoflagellate neurotoxin, phosphatase inhibitor
I-606061 Up	5.38E-21	synthetic anti-proliferative drug, unknown mechanism
Bufalin Up	7.83E-20	toad toxin, induction of cell cycle arrest
QL-XII-47 Up	4.26E-19	synthetic translation inhibitor
Cercosporin Up	3.20E-18	Cercosporin toxin, oxidative stressor
NSC-663284 Up	3.81E-18	synthetic CDC25 phosphatase inhibitor
I-606051 Up	2.12E-17	synthetic anti-proliferative drug, unknown mechanism
Digitoxigenin Up	3.44E-17	plant glyoside, ATPase inhibitor

T120 up

Term	Adj. P-value	description of LINCS 1000 drug
Narciclasine Up	3.70E-37	plant alkaloid, translation inhibitor
Anisomycin Up	8.67E-33	Streptomyces toxin, translation-elongation inhibitor
NSC-663284 Up	1.24E-23	synthetic CDC25 phosphatase inhibitor
Cercosporin Up	4.43E-23	Cercosporin toxin, oxidative stressor
QL-XII-47 Up	4.43E-23	synthetic translation inhibitor
Helveticoside Up	8.09E-23	flixweed glycoside with unknown target
Salermide Up	9.20E-20	synthetic sirtuin inhibitor
I-606061 Up	9.20E-20	synthetic anti-proliferative drug, unknown mechanism
I-606051 Up	1.17E-19	synthetic anti-proliferative drug, unknown mechanism
Okadaic-Acid Up	2.53E-19	dinoflagellate neurotoxin, phosphatase inhibitor

T24 up

Term	Adj. P-value	description of LINCS 1000 drug
Emetine Up	1.16E-06	ipecacuanha toxin, translation-elongation inhibitor - emetic
Narciclasine Up	5.39E-06	plant alkaloid, translation inhibitor
Cephaeline Up	4.73E-05	ipecac alkaloid, translation-elongation inhibitor - emetic
Anisomycin Up	5.29E-05	Streptomyces toxin, translation-elongation inhibitor
Bruceantin Up	6.18E-04	Brucea triterpene, translation-elongation inhibitor
I-606061 Up	0.004	synthetic anti-proliferative drug, unknown mechanism
Amanitin Down	0.007	inhibitor of RNA PolII, but not RNA PolI; i.e. ribosomal RNA is transcribed
Emetine-Hcl Up	0.009	ipecacuanha toxin, translation-elongation inhibitor - emetic
QL-XII-47 Up	0.009	synthetic translation inhibitor
I-606051 Up	0.009	synthetic anti-proliferative drug, unknown mechanism

T15 down

Term	Adj. P-value	description of LINCS 1000 drug
Clocortolone Up	0.372	
PSB-36 Up	0.372	
Androstenedione Down	0.372	
LIVF001-022 Up	0.372	
Vortioxetine Up	0.372	
LY-344864 Up	0.372	
Tropicamide Down	0.372	
Caprolactam Down	0.372	
PNU-282987 Up	0.372	
YM-155 Down	0.372	

T30 down

Term	Adj. P-value	description of LINCS 1000 drug
Trimethobenzamide Up	0.372	
Miglitol Down	0.372	
Diallyl-Phthalate Down	0.372	
Quercetin Down	0.372	
LY-2183240 Down	0.372	
Retinyl Up	0.372	
GALR2 GALANIN Up	0.372	
Fexofenadine Up	0.372	
VU-0402604 Up	0.372	
Diethylcarbamazine Down	0.372	

T60 down

Term	Adj. P-value	description of LINCS 1000 drug
2-BFI Down	0.403	
Almotriptan Down	0.403	
Metacetamol Down	0.403	
Valproic-Acid Down	0.403	
Cardiogenol-C Down	0.403	
Cyclopentene Down	0.403	
DMeOB Down	0.403	
TUL-XX025TFA Down	0.403	
Chloroxylenol Down	0.403	
Nifenazone Up	0.403	

T90 down

Term	Adj. P-value	description of LINCS 1000 drug
WR-216174 Up	0.581	
Lanatoside-C Down	0.581	
Peruvoside Down	0.581	
Bufalin Down	0.581	
LFM-A13 Down	0.581	
Benazepril Down	0.581	
KDM-103 Down	0.581	
ST-012015 Up	0.581	
Fumonisin-B1 Down	0.581	
Nifekalant Down	0.581	

T120 down

Term	Adj. P-value	description of LINCS 1000 drug
Flumethasone-Pivalate Up	0.181	
Narciclasine Down	0.754	
8-M-PDOT Up	0.754	
PI-103 Up	0.754	
4-ACETYLAMINOBIPHENYL Down	0.754	
CHIR-99021 Down	0.754	
LY-2606368 Down	0.754	
Isoflupredone-Acetate Up	0.754	
PF-477736 Down	0.754	
Novobiocin Up	0.754	

T24 down

Term	Adj. P-value	description of LINCS 1000 drug
Obatoclax Up	1.85E-13	synthetic Bcl2 inhibitor, pro-apoptotic
Orlistat Up	3.18E-12	synthetic lipase inhibitor
Cycloheximide Down	3.83E-12	Streptomyces translation inhibitor
Tandutinib Up	4.78E-12	synthetic tyrosine kinase inhibitor
Ceritinib Up	4.78E-12	synthetic kinase inhibitor
Salmeterol Up	4.78E-12	synthetic β2 adrenergic receptor agonist
SRT-1720 Up	4.78E-12	synthetic sirtuin activator
VU-0155069 Up	4.78E-12	synthetic phospholipase D1 inhibitor
PD-173074 Up	4.93E-12	synthetic FGFR inhibitor
Lovastatin Up	1.25E-11	synthetic HMG-CoA reductase inhibitor

Supplementary table 10: Similarities of gene expression changes in V79 cells treated with DON to other transcriptome datasets in the LINCS 1000 project. The genes changed significantly (P<0.05; fold change >2) in V79 cells in response to DON treatment for 15 min. (T15), 30 min. (T30), 60 min. (T60), 90 min. (T90), 120 min. (T120) or 24 hours (T24) were analysed in the EnrichR software suite (https://maayanlab.cloud/Enrichr/). DON responsive gene expression profiles were compared to available cell transcriptomes characterized in response to treatment with defined chemicals. The top 10 transcriptome profiles identified for the different treatment periods are shown (genes upregulated in response to DON treatment are shown on left hand side, and genes downregulated in response to DON treatment are shown on right hand side for the 6 experimental timepoints) together with the p-values of the transcriptome similarities. Chemical treatments are ordered by p-values. Chemicals which elicit transcriptome changes similar to DON across all experimental timepoints are marked in identical colours in the first column of the table on the left-hand side (upregulated genes). Chemicals which are known inhibitors of translation are marked in blue in the 3rd column in both sides of the table.

gene	IC50 [ng/ml]	LOD [ng/ml]
Jun	124	30
ATF3	196	30
Fos	863	30
H2B	599	125
H2A	2317	125

Supplementary table 11: IC50 values and LOD (limits of detection) values (in ng/ml) for the expression of DON responsive genes in V79 Chinese Hamster lung fibroblasts. V79 cells were treated with DON at concentrations ranging from 2000ng/ml to 0.03ng/ml. Expression rates for Jun, ATF3, Fos, and histones H2A-2a and H2B-1c were measured by qPCR (n=4).

Supplementary figures

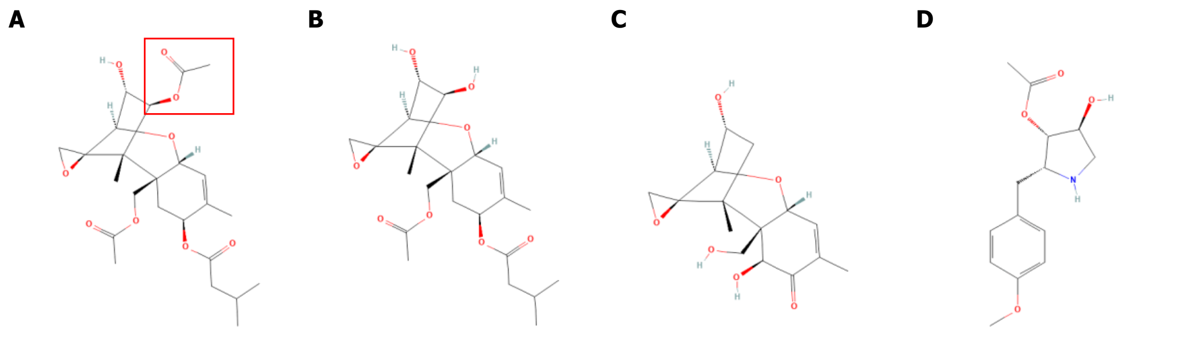

T2 toxin
MW: 466.5 g/mol
SMILES:
CC1=C[C@@H]2[C@](C[C@@H]1OC(=O)CC(C)C)([C@]3([C@@H]([C@H]([C@H]([C@@]34CO4)O2)O)OC(=O)C)C)COC(=O)C

HT2 toxin
MW: 424.5 g/mol
SMILES:
CC1=C[C@@H]2[C@](C[C@@H]1OC(=O)CC(C)C)([C@]3([C@@H]([C@H]([C@H]([C@@]34CO4)O2)O)O)C)COC(=O)C

deoxynivalenol
MW: 296.31 g/mol
SMILES:
CC1=C[C@@H]2[C@]([C@@H](C1=O)O)([C@]3(C[C@H]([C@H]([C@@]34CO4)O2)O)C)CO

anisomycin
MW: 265.30 g/mol
SMILES:
CC(=O)O[C@@H]1[C@H](CN[C@@H]1CC2=CC=C(C=C2)OC)O

Supplementary figure 1: Chemical structure, molecular weight and SMILES formula for the toxins T2 (**Panel A**), HT2 (**Panel B**), deoxynivalenol (**Panel C**), and anisomycin (**Panel D**).

A

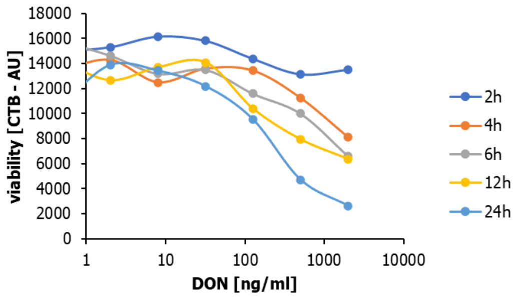

B

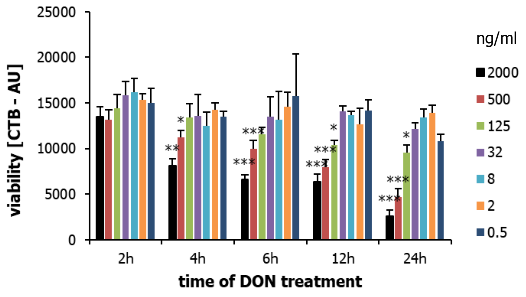

Supplementary figure 2: Impact of DON exposure on V79 cells over time. **Panel A:** Viability of V79 cells measured after exposure of V79 cells to DON concentrations from 1ng/ml to 2000ng/ml. Viability is shown as arbitrary units (AU) read in a Cell Titre Blue assay. **Panel B:** ANOVA analysis with Bonferroni post-hoc test of viability changes relative to control cells (n=4). p<0.05 *; p<0.01 **; p<0.001 ***.

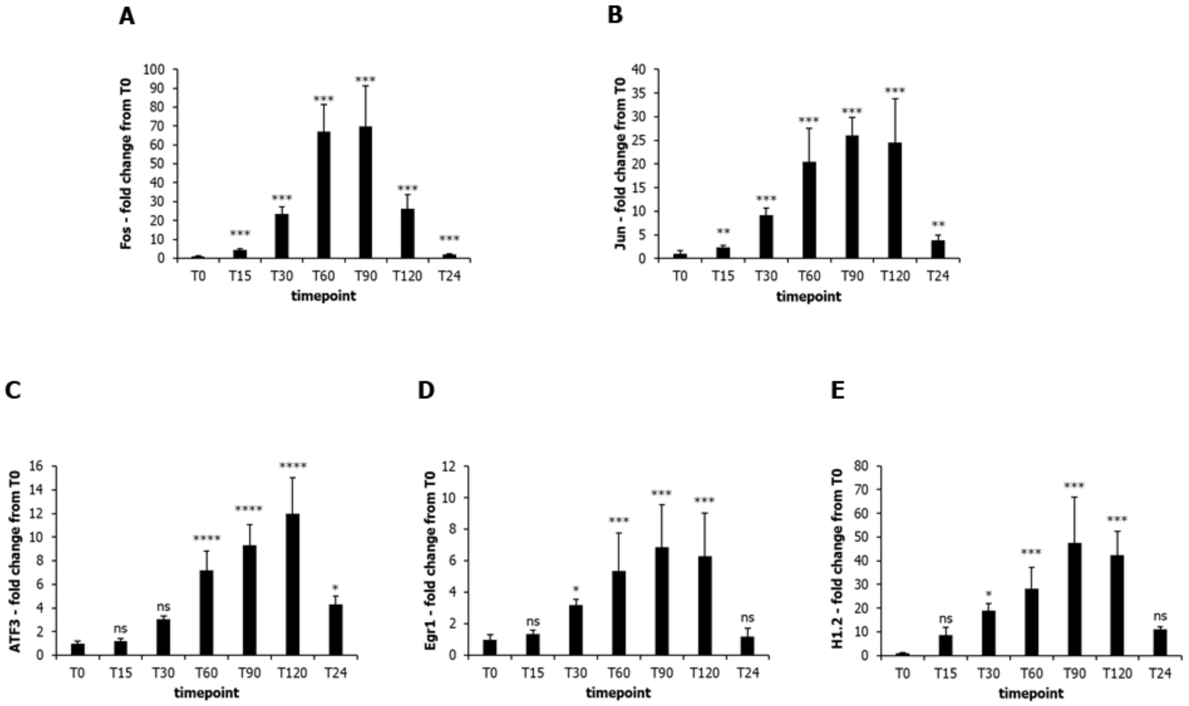

Supplementary figure 3: qPCR confirmation of RNAseq data. cDNA samples derived from V79 cells exposed to 500ng/ml of DON for 0 min. (T0, control), 15 min. (T15), 30 min. (T30), 60 min. (T60), 90 min. (T90), 120 min. (T120) or 24 hours (T24) were analysed using gene specific primer pairs for Fos (**Panel A**), Jun (**Panel B**), ATF3 (**Panel C**), Egr1 (**Panel D**), and histone H1.2 (**Panel E**) and the control gene β-actin. Expression of the regulated genes was correlated with the expression of β-actin and is shown as fold change in mRNA expression from the control value (T0) (n=4). Data were analysed by one way ANOVA with a Bonferroni post-hoc test in GraphPad Prism. P<0.05: *; p<0.01: **; p<0.001: ***.

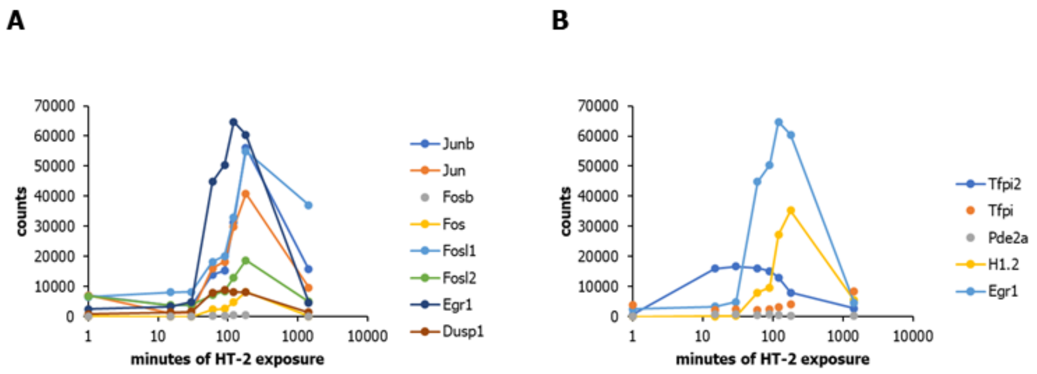

Supplementary figure 4: Gene expression response of V79 cells to treatment with the trichothecene mycotoxin HT2. **Panel A:** Expression of the stress response transcription factors Fos, Jun, Egr1 and Dusp1 over the treatment period (timepoints T0, T15, T30, T60, T90, T120, T180, T24). **Panel B:** Expression of the transcription factor Egr1, the histone gene H1.2 and the enzyme coding genes Tfpi1 (tissue factor pathway inhibitor) and PDEA2 (phosphodiesterase 2) over treatment timepoint. Note that the peak of stress response transcription factors occurs around 30 minutes later than in V79 cells exposed to DON.

A

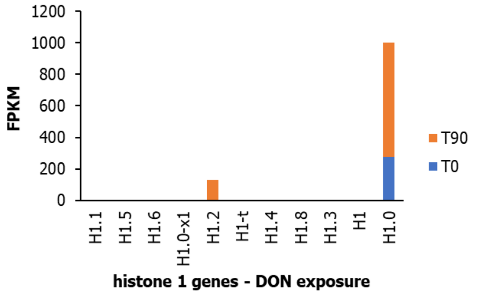
1200
1000
800
600
400
200
0
FPKM
T90
T0
H1.1
H1.5
H1.6
H1.0-x1
H1.2
H1-t
H1.4
H1.8
H1.3
H1
H1.0
histone 1 genes - DON exposure

B

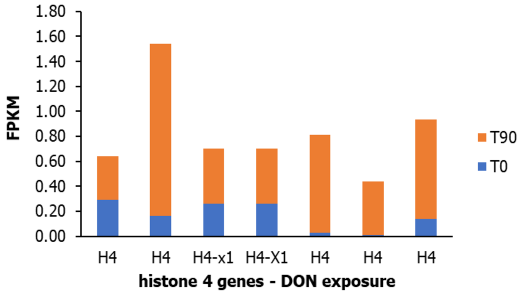
1.80
1.60
1.40
1.20
1.00
0.80
0.60
0.40
0.20
0.00
FPKM
T90
T0
H4
H4
H4-x1
H4-X1
H4
H4
H4
histone 4 genes - DON exposure

C

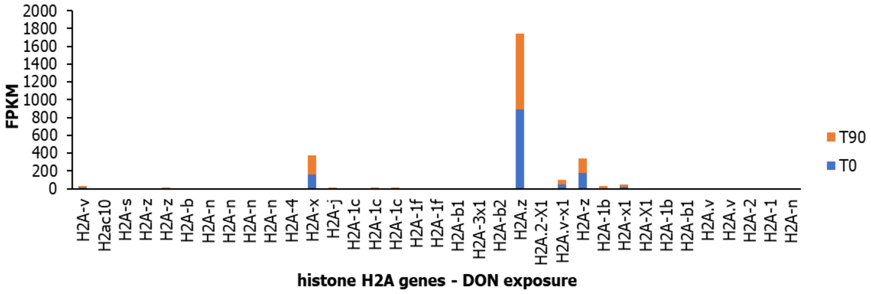
2000
1800
1600
1400
1200
1000
800
600
400
200
0
FPKM
T90
T0
H2A-v
H2ac10
H2A-s
H2A-z
H2A-z
H2A-b
H2A-n
H2A-n
H2A-n
H2A-n
H2A-4
H2A-x
H2A-j
H2A-1c
H2A-1c
H2A-1c
H2A-1f
H2A-1f
H2A-b1
H2A-3x1
H2A-b2
H2A.z
H2A.2-X1
H2A.v-x1
H2A-z
H2A-1b
H2A-x1
H2A-X1
H2A-1b
H2A-b1
H2A.v
H2A.v
H2A-2
H2A-1
H2A-n
histone H2A genes - DON exposure

D

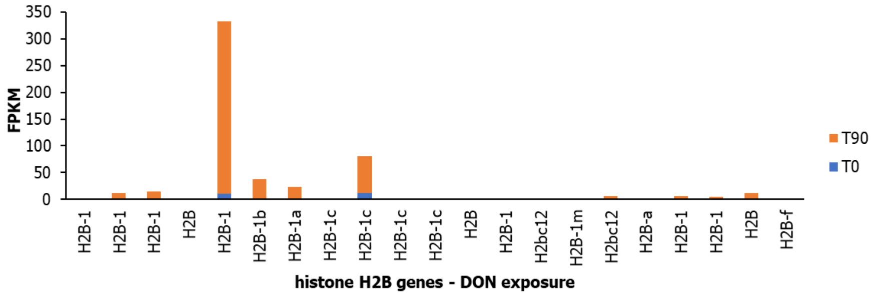

E

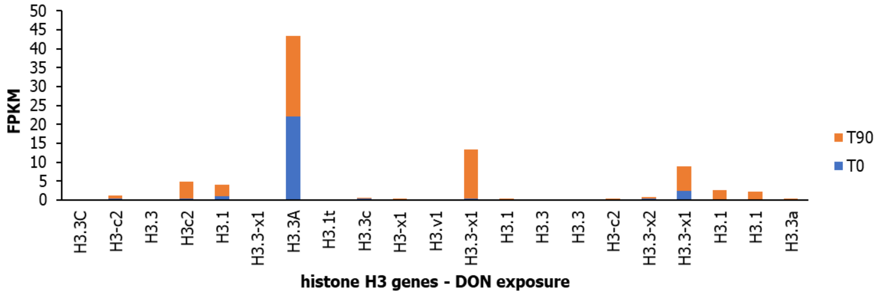

Supplementary figure 5. Histone gene expression in response to DON exposure. Histone gene expression (in FPKM) at T0 (control cells) is shown as the blue part of the bar; expression at T90 (90 minutes of DON exposure) is shown as the orange part of the bar. If the orange and blue part of the bar are of equal size, DON exposure does not alter gene expression. **Panel A:** Expression of all (identifiable) histone 1 genes at timepoints T0 (prior to DON exposure) and T90 (at the maximum gene expression response to DON exposure). Histones H1.0 and histone H1.2 are the predominant H1 histones, and both are significantly increased at time point T90. **Panel B:** Expression of all histone H4 genes at timepoints T0 and T90. Transcription of all H4 histone genes is significantly increased at time point T90. **Panel C**: Expression of all histone H2A genes at timepoints T0 and T90. Note that the most highly expressed H2A histones are already substantially expressed at time point T0 and are only activated by around 2-fold in response to DON exposure. **Panel D:** Expression of all histone H2B genes at timepoints T0 and T90. Note that histone H2B gene expression is much higher at timepoint T90 than at timepoint T0. **Panel E:** Expression of all histone H3 genes at timepoints T0 and T90.

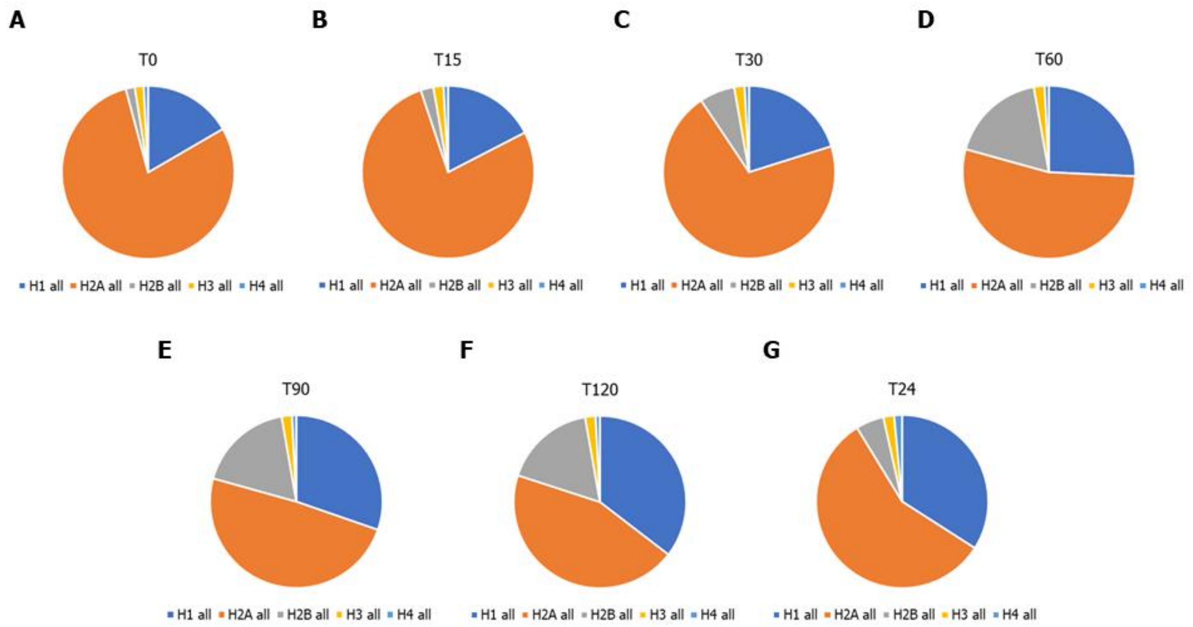

Supplementary figure 6. Pie chart of expression of different classes of histone genes in V79 cells exposed to 500ng/ml of DON for 15 min. (**Panel B:** T15), 30 min. (**Panel C:** T30), 60 min. (**Panel D:** T60), 90 min. (**Panel E:** T90), 120 min. (**Panel F:** T120) and 24 hours (**Panel G:** T24). Expression in the absence of DON treatment is shown in **Panel A** (T0). H1: histone 1; H2A: histone H2A; H2B: histone H3; H4: histone H4.

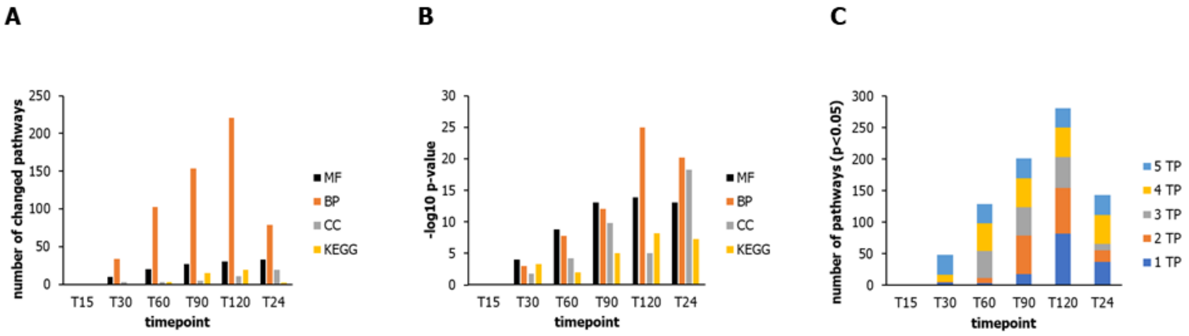

Supplementary figure 7. Pathway analysis of transcriptome changes at the different timepoints using the G-Profiler platform. **Panel A:** Number of pathways significantly changed (relative to control cells at timepoint T0) in response to DON exposure at different timepoints. Pathways identified are for GO-terms of "molecular function" (MF), "biological process" (BP), "cellular compartment" (CC) and the KEGG pathways (KEGG). Note that no significantly changed pathways are detected at timepoint T15 (15 minutes exposure of V79 cells to DON). **Panel B:** P-values of the pathways significantly changed in response to DON exposure at the different timepoints of the study (shown as -log10 p-values). **Panel C:** Similarity of pathways changed at different time pints. The GO terms of significantly changed pathways were compared by a Venn analysis. Significantly changed pathways at all 5 timepoints (5 TP), 4 timepoints (4 TP), 3 timepoints (3 TP), or 2 time points (2 TP) are shown. Significantly changed pathways which are unique to a specific timepoint are shown as 1 TP. Note that most of the early pathway changes are maintained throughout the incubation period, but unique responses for that time point develop once bigger transcriptional changes are generated by the toxin treatment (from T90 onwards).

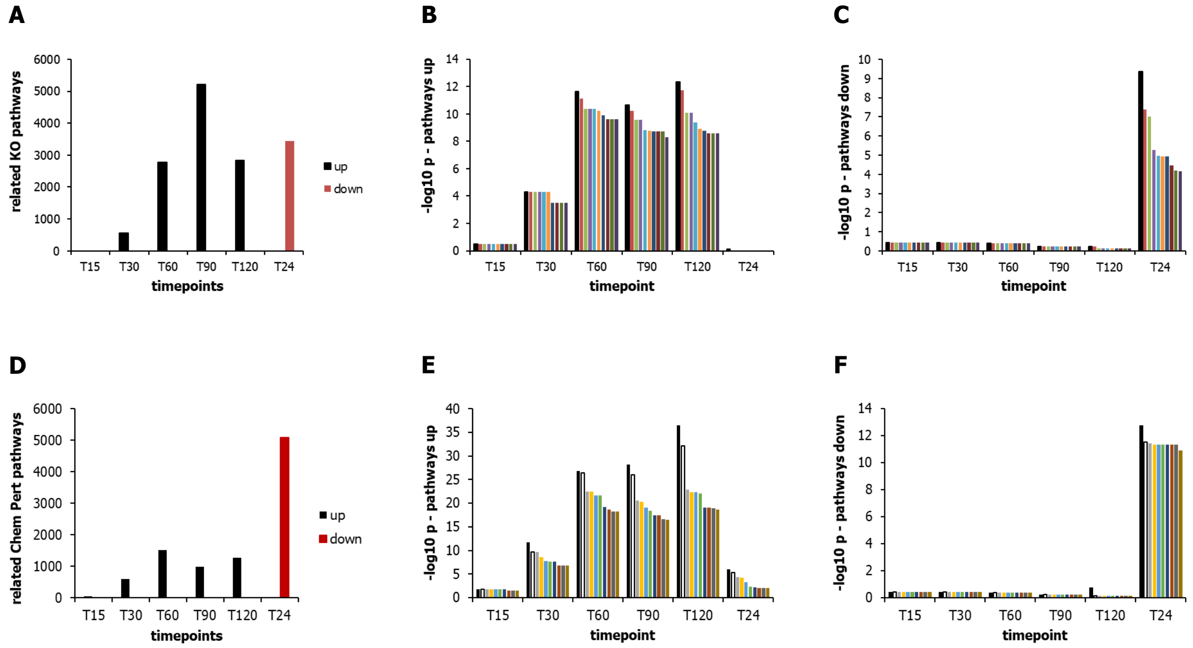

Supplementary figure 8: Similarities of gene expression changes in V79 cells treated with DON to other transcriptome datasets in the LINCS 1000 project. The genes changed significantly ($P<0.05$; fold change >2) in V79 cells in response to DON treatment for 15 min. (T15), 30 min. (T30), 60 min. (T60), 90 min. (T90), 120 min. (T120) or 24 hours (T24) were analysed in the EnrichR software suite (https://maayanlab.cloud/Enrichr/). DON responsive gene expression profiles were compared to available CRISPR knock-out cell transcriptomes (**Panels A-C**) and transcriptomes induced by chemical treatment (**Panels D-F**). **Panel A:** Number of CRISPR-knock-out cell lines in which transcriptome changes (relative to the parental cell lines) significantly overlap with those induced by DON treatment. **Panel B:** -log10 p-values of the top 10 gene knockouts which generate transcriptome changes which significantly overlap with those induced by DON treatment. The p-values for genes upregulated in response to DON treatment are shown. **Panel C:** -log10 p-values of the top 10 gene knockouts which generate transcriptome changes which significantly overlap with those induced by DON treatment. The p-values for genes downregulated in response to DON treatment are shown. **Panel D:** Number of chemical-treated cell lines for which transcriptome changes (relative to untreated control cells) significantly overlap with those induced by DON treatment. **Panel E:** -log10 p-values of the top 10 chemical treatments which generate transcriptome changes which significantly overlap with those induced by DON treatment. The p-values for genes upregulated in response to DON treatment are shown. **Panel F:** -log10 p-values of the top 10 chemical treatments which generate transcriptome changes which significantly overlap with those induced by DON treatment. The p-values for genes downregulated in response to DON treatment are shown.
